# Research status of east Asian traditional medicine treatment for chronic cough: A scoping review

**DOI:** 10.1371/journal.pone.0296898

**Published:** 2024-02-08

**Authors:** Boram Lee, Chan-Young Kwon, Ye Ji Kim, Jae Hyun Kim, Kwan-Il Kim, Beom-Joon Lee, Jun-Hwan Lee

**Affiliations:** 1 KM Science Research Division, Korea Institute of Oriental Medicine, Daejeon, South Korea; 2 Department of Oriental Neuropsychiatry, Dong-eui University College of Korean Medicine, Busan, South Korea; 3 Department of Korean Pediatrics, Kyung Hee University Medical Center, Seoul, South Korea; 4 Department of Korean Pediatrics, Kyung Hee University Hospital at Gangdong, Seoul, South Korea; 5 Department of Internal Medicine, Division of Allergy, Immune and Respiratory System, College of Korean Medicine, Kyung Hee Medical Center, Kyung Hee University, Seoul, South Korea; 6 Department of Korean Convergence Medical Science, KIOM School, University of Science & Technology (UST), Daejeon, South Korea; 7 Department of Radiology, Athinoula A. Martinos Center for Biomedical imaging, Massachusetts General Hospital, Harvard Medical School, Boston, MA, United States of America; Kaohsuing Medical University Hospital, TAIWAN

## Abstract

**Background:**

When patients continue to experience cough despite conventional treatment, East Asian traditional medicine (EATM) including herbal medicine and/or acupuncture has been frequently used. Previous systematic reviews of EATM treatment for chronic cough have been conducted mainly on herbal medicine, targeting patients with conditions that cause cough. In clinical practice, EATM interventions are not limited to herbal medicine, and considering that chronic cough is often caused by two or more conditions or unspecific causes, a comprehensive investigation is clinically relevant. We examined the current research status of EATM for chronic cough.

**Methods:**

Based on Arksey and O’Malley’s scoping review methodological framework, a total of six English, Chinese, Korean, and Japanese electronic databases were searched on August 2022. Any clinical studies on EATM targeting chronic cough patients (regardless of their cause) were included.

**Results:**

Among 474 included studies, the study designs were mainly randomized controlled trials (72.4%), and the population was evenly distributed between children and adults. The cause of cough was not reported in most studies (56.1%). The common cause of cough was upper airway cough syndrome and post-respiratory infection (9.5%, each), followed by mixed cause (7.6%), nonspecific cause (5.9%), and gastroesophageal reflux disease (4.0%). EATM was conducted for a mean of 19.1 days, and herbal medicine was the most common (80.6%). Conventional medication was frequently used as a control (81.2%). For outcomes, the total effective rate was the most frequently utilized (94.3%), followed by cough severity (53.8%). EATM treatment showed positive outcomes in most studies.

**Conclusions:**

In future EATM studies, it is necessary to either specify the cause of chronic cough or to report that the study was targeting nonspecific chronic cough. In addition, high-quality studies assessing the efficacy of EATM with placebo control treatment should be conducted, using validated evaluation tools.

## Introduction

Chronic cough is defined as a cough lasting more than eight weeks in adults and more than four weeks in children younger than 15 years [1, 2]. Currently, the prevalence of chronic cough is high, exceeding 10% of the total population, and 14–23% of adult non-smokers have chronic cough [3]. As chronic cough is ubiquitous, especially in the elderly population, the disease burden of chronic cough is expected to increase with a rapid population-aging trend, resulting in a significant socioeconomic burden [4]. In severe cases of cough, complications such as nausea, chest pain, urinary incontinence, and depression may arise [5]. In addition, patients with chronic cough reported poor health-related quality of life, impairment of work productivity and activity, and utilization of more healthcare resources [6]. Typical causes of chronic cough include upper airway cough syndrome (UACS), asthma including cough variant asthma (CVA), and gastroesophageal reflux disease (GERD) [7]. However, more than one condition may be involved at a time. There are also a significant number of patients with nonspecific chronic cough (with no identifiable cause after a reasonable evaluation, including history taking, physical examination, radiography, and spirometry), and unexplained chronic cough (idiopathic or refractory; with an unknown etiology that persists after a comprehensive investigation, medical assessment, and appropriate therapeutic trials) [8–11].

The conventional treatment of chronic cough involves the administration of medications to treat the cause of the cough, and empirical treatment with antitussives, expectorants, or mucolytics is provided when it is difficult to accurately test or differentiate. However, 10–40% of patients with chronic cough who visit advanced medical institutions are not treated appropriately despite the best diagnostic tests and treatment efforts [12]. In addition, the effect is limited in more than 70% of patients with chronic cough, which may be mainly attributed to the insignificant effect of conventional treatments and equivocal diagnostic methods [13]. Therefore, an effective alternative treatment approach that can be applied even when the cause is unclear or when the cough is resistant to conventional treatments is required. In this situation, East Asian traditional medicine (EATM) treatment including herbal medicine and/or acupuncture has long been used for cough [14, 15]. However, despite its utilization [14, 15], clinical evidence has not been sufficiently accumulated, raising concerns about its effectiveness and safety [16]. Previous systematic reviews of EATM treatment for chronic cough have been conducted mainly on herbal medicine treatment, targeting patients with conditions that cause cough, such as UACS and CVA [17–19]. However, no attempt has been made to comprehensively investigate clinical studies of EATM interventions besides herbal medicine for chronic cough, regardless of its cause. In clinical practice, because EATM interventions are not limited to herbal medicine, and considering that chronic cough is often caused by two or more conditions or unspecific causes, a comprehensive investigation is clinically relevant.

A scoping review summarizes the scope and number of available literature articles and examines the nature and content of the research evidence, aiming to quickly map the main concepts, primary sources of information, and types of available evidence supporting the research area [20]. It does not describe individual research results in detail. It is conducted to investigate the scope of the literature of a specific area, identify the populations, interventions, comparators, and outcomes (PICOs), and investigate the potential subject range [21]. Therefore, we used a scoping review methodology to examine the current clinical research status of various EATM treatments for chronic cough regardless of its cause by analyzing study designs, populations, EATM treatments, and outcome measures used in existing clinical studies and proposed appropriate PICOs for future systematic reviews of EATM.

## Methods

We conducted the study according to the following 5-step scoping review protocol based on the Arksey and O’Malley framework [20, 22]: 1) Identifying the research question, 2) Identifying relevant studies, 3) Study screening and selection, 4) Data extraction, and 5) Collating, summarizing, and reporting the results. This study was described according to the Preferred Reporting Items for Systematic Reviews and Meta-Analyses Extension for Scoping Reviews Checklist and Explanation [23], and the study protocol was registered in the Open Science Framework (OSF) registry prior to the study (URL: https://osf.io/7w3q4).

### Identifying the research question

As described in the Introduction section, the purpose of this scoping review was to evaluate the status of clinical studies of various EATM treatments for chronic cough regardless of its cause. Therefore, the research question in this scoping review was as follows: *“How much clinical research on EATM treatments has been conducted for chronic cough*, *and which study designs (i*.*e*., *case report*, *case series*, *non-randomized controlled clinical trial [CCT]*, *and randomized controlled clinical trial [RCT])*, *population (i*.*e*., *the cause of cough and age [children and adults])*, *EATM treatment (herbal medicine*, *acupuncture*, *combination treatment*, *etc*.*)*, *and outcome measures were used*?”

### Identifying relevant studies

#### 1) Information sources

The following six databases were searched on August 21, 2022, by one researcher (BL): three English databases (Medline, EMBASE, and Cochrane Central Register of Controlled Trials), one Chinese database (China National Knowledge Infrastructure), one Korean database (Oriental Medicine Advanced Searching Integrated System), and one Japanese database (CiNii). All retrieved studies from each database from their inception dates to the search date were reviewed. To identify potentially missing literature, the reference lists of studies included in the scoping review were also screened. The database search strategy combined chronic cough corresponding to the population and EATM treatment corresponding to the intervention. The search terms were a combination of index words and free words and were finalized after discussions with specialists in respiratory medicine (K.I.K. and B.J.L.) and systematic review methodology (C.Y.K.). Detailed search strategies and the results for each database are presented in S1 Appendix.

#### 2) Eligibility criteria

(1) Populations: Studies targeting patients with chronic cough (i.e., cough lasting more than eight weeks in adults aged ≥ 15 years and four weeks in children aged < 15 years [1, 2]) regardless of sex, age, race, and the cause of cough were included. However, studies targeting patients with comorbid diseases other than chronic cough or studies that did not specify the duration of cough were excluded.

(2) Interventions/comparators: For therapeutic interventions, treatments based on EATM including herbal medicine, acupuncture, moxibustion, cupping therapy, chuna, pharmacopuncture, and a combination of them were included. As this review focused on herbal compositions formulated according to EATM, studies of single herbs were excluded. In cases where EATM treatment and conventional treatment were used together as medical interventions in controlled studies, only studies using the same conventional treatment as the control intervention were included. There were no other limitations on the comparators. If treatments were based on the EATM theory, no restrictions were placed on the country of publication.

(3) Outcomes: There were no restrictions on outcome variables.

(4) Study designs: Original human subject clinical studies such as RCTs, CCTs, retrospective cohort studies, before-after studies, case series, and case reports were included. Study protocols, review articles, and non-clinical studies were excluded.

(5) Others: There were no restrictions on language, publication year, and publication type such as journal articles, theses, and conference proceedings. However, we excluded preprints under review.

### Study screening and selection

The study screening and selection process were performed using EndNote 20 (Clarivate Analytics, Philadelphia, PA, USA) for studies retrieved from database searches and other data sources. First, after removing duplicate entries using the duplicate literature removal function of EndNote 20, eligible studies were included after title and abstract review based on the eligibility criteria. Then, the full texts of eligible literature articles were obtained, and reviewed for final inclusion in the literature sources. Two researchers (B.L. and C.Y.K.) conducted the study screening and selection independently, and in cases of disagreement, an agreement was reached through discussion between the same two researchers.

### Data extraction

Data extraction was independently performed by four researchers (B.L., C.Y.K., Y.J.K., and J.H.K.) using pilot-tested Excel forms, and discrepancies were resolved through discussions amongst themselves. The data extraction form was pilot-tested by two authors (B.L. and C.Y.K.) who independently extracted items from three included studies, which was developed into a comprehensive form that included all necessary but missing items.

From the final included studies, the following information was extracted using a pilot-tested Excel form: year of publication, study design, number of patients/participants included, country and language of publication, study setting, study population (children/adults, causes of chronic cough), types of EATM treatment (such as herbal medicine, acupuncture, and moxibustion), control group intervention (in the case of controlled studies), treatment period, outcome measures, and results. We classified the study designs of included studies according to the design algorithm for studies of interventions and exposures [24]. For outcome measures, the following indicators were extracted: (1) Cough severity assessed by scales such as cough symptom score (CSS), visual analog scale (VAS), and traditional Chinese medicine symptom score; (2) cough frequency; (3) cough-related quality of life assessed by scales such as the Leicester cough questionnaire (LCQ) and cough-specific quality-of-life questionnaire (CQLQ); (4) total effective rate (TER); (5) blood biomarkers such as eosinophil counts and C-reactive protein; (6) pulmonary function test indices such as forced expiratory volume in one second and forced vital capacity; and (7) adverse events. As this scoping review was conducted to provide an overview of existing studies in this field, the results reported in individual studies were out of scope and were not extracted. Instead, we extracted and classified the effect of EATM on each outcome measure in individual studies as “Positive outcomes”, “Not different from a control” (applies only to controlled studies), and “Inconsistent” (inconsistent results among assessment tools that evaluate the same outcome measure [e.g., in cough severity assessment, EATM showed positive outcomes with VAS but not CSS]). Adverse events were extracted only to determine whether they were reported in the included studies.

### Collating, summarizing, and reporting the results

Descriptive analysis was performed on the extracted data, and the results are expressed as the frequency and ratio for categorical variables and the mean and standard deviation (SD) for continuous variables. As the scoping review did not evaluate the methodological quality of the included studies and minimized data synthesis, the results of the current analysis are presented using figures and tables through a descriptive and quantitative summary. All statistical analyses were performed using Excel 2019 (Microsoft Office, Redmond, WA, USA).

## Results

### Study selection and study characteristics

**Fig 1:**
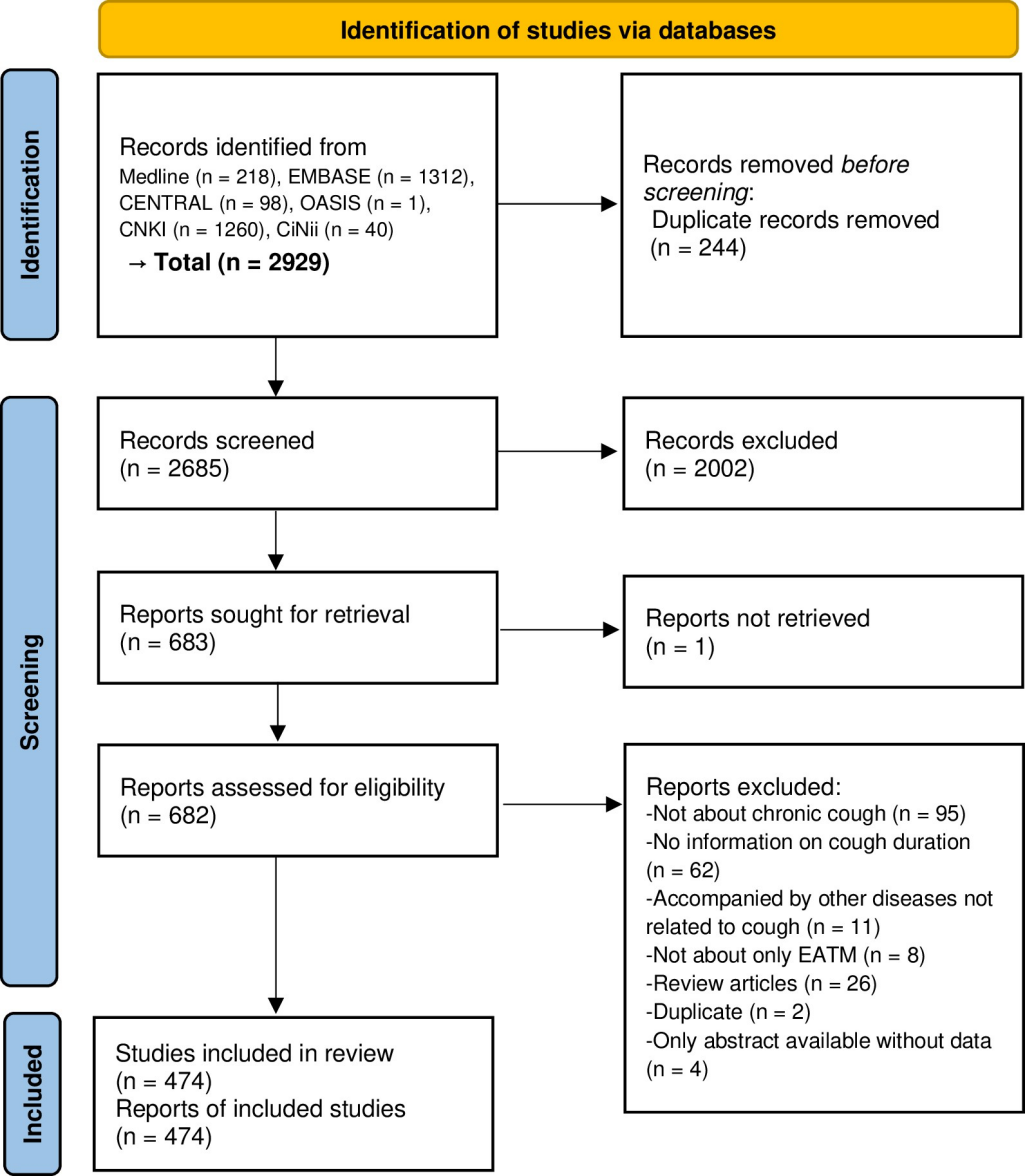
Study selection process. No additional studies were identified from the reference lists of studies included in the scoping review. Abbreviation. EATM, East Asian traditional medicine.

**Fig 2:**
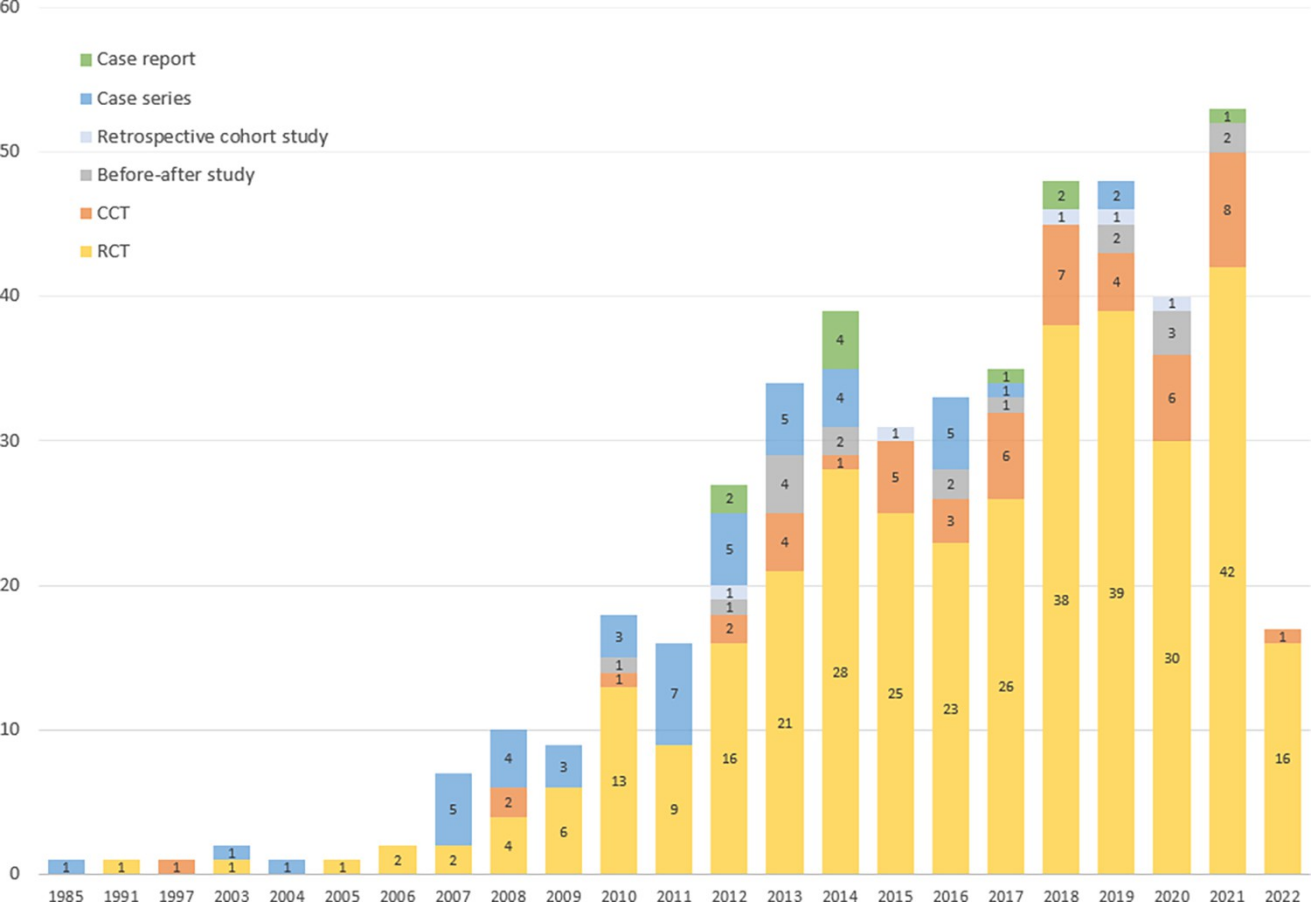
Research status according to the publication year. Abbreviations. CCT, non-randomized controlled clinical trial; RCT, randomized controlled clinical trial.

A total of 2929 articles were searched from the databases. After removing 244 duplicate articles, the titles and abstracts of 2685 articles were screened, and 2002 articles were excluded. Subsequently, 682 studies for which full-texts could be confirmed were reviewed, and 208 studies were excluded for the following reasons: not about chronic cough (n = 95), no information on cough duration (n = 62), accompanied by other diseases not related to cough (n = 11), not only for EATM (n = 8), review articles (n = 26), duplicate (n = 2), and only abstracts available without data (n = 4) (S2 Appendix). Finally, a total of 474 articles were included (Fig 1 and S3 Appendix). In terms of publication year, the number of included studies increased sharply after 2012. In terms of study designs, before 2011, the proportion of observational studies including case series, case reports, and retrospective cohort studies was similar to that of interventional studies including RCTs and CCTs; however, after 2012, the number of interventional studies was markedly augmented (Fig 2).

In terms of study designs, 343 (72.4%) RCTs accounted for a majority of studies. There were 51 (10.8%) CCTs, 18 (3.8%) before-after studies, five (1.1%) retrospective cohort studies, 47 (9.9%) case series, and 10 (2.1%) case reports (Table 1). The number of study subjects per study was 94.6 ± 75.4 (mean ± SD) (range, 1~663). The number of subjects according to the study design was 99.1 ± 75.3 (range, 24~663) for RCTs, 85.6 ± 34.3 (range, 36~200) for CCTs, 99.5 ± 73.8 (range, 30~300) for before-after studies, 92.0 ± 32.0 (range, 60~124) for retrospective cohort studies, 91.8 ± 88.9 (range, 30~450) for case series, and 1.3 ± 0.7 (range, 1~3) for case reports.

**Table 1:** General and demographic characteristics of the included studies.

Variables	Categories	N	%
Study design	Case report	10	2.1%
	Case series	47	9.9%
	Retrospective cohort study	5	1.1%
	Before-after study	18	3.8%
	CCT	51	10.8%
	RCT	343	72.4%
Country	China	473	99.8%
	South Korea	1	0.2%
Study setting	Hospitals/clinics	471	99.4%
	Public health center	1	0.2%
	Not reported	2	0.4%
Study population	Children	172	36.3%
	Adults	231	48.7%
	Both	67	14.1%
	Not reported	4	0.8%
Cause of cough	UACS	45	9.5%
	CVA	10	2.1%
	GERD	19	4.0%
	Chronic bronchitis	5	1.1%
	Eosinophilic bronchitis	2	0.4%
	Post-respiratory infection	45	9.5%
	Nonspecific	28	5.9%
	Unexplained	7	1.5%
	Mixed cause	36	7.6%
	Others*	11	2.3%
	Not reported	266	56.1%
Total		474	100.0%

*including bronchiectasis, interstitial pulmonary fibrosis, allergic cough, angiotensin-converting enzyme inhibitors-induced cough, psychogenic cough, airway hyperresponsiveness, chronic obstructive pulmonary disease, cough after lung cancer surgery, pulmonary tuberculosis, chronic sore throat, etc.

Abbreviations. CCT, non-randomized controlled clinical trial; CVA, cough variant asthma; GERD, gastroesophageal reflux disease; RCT, randomized controlled clinical trial; UACS, upper airway cough syndrome.

In terms of country of publication, 473 (99.8%) studies were published in China, and only one (0.2%) RCT was published in South Korea. In terms of publication language, 472 (99.6%) articles were published in Chinese, and the remaining two (0.4%) articles were published in English. In terms of study settings, 471 (99.4%) studies were conducted in hospitals or clinics. Only one (0.2%) study was conducted in a public health center, and two (0.4%) studies did not report the study setting (Table 1).

### Demographic characteristics of the study populations

Among the studies describing the cause of cough, UACS (45 studies, 9.5%), post-respiratory infection (45 studies, 9.5%), mixed cause (studies involving patients with more than one cause of cough, such as both UACS and CVA) (36 studies, 7.6%), and nonspecific (28 studies, 5.9%) were the most frequently reported. Most studies (266 studies, 56.1%) did not state the cause of chronic cough (Table 1). Analysis according to the age of the study population revealed that post-respiratory infection (37 studies, 21.5%) was most frequent in studies involving children, followed by UACS (29 studies, 16.9%) and mixed cause (13 studies, 7.6%), excluding studies that did not report the cause of cough. However, in studies involving adults, mixed cause (17 studies, 7.4%) was the most ordinary, followed by nonspecific (15 studies, 6.5%) and GERD (13 studies, 5.6%). In the case of cough due to UACS and post-respiratory infection, more than 50% of studies involved children. On the other hand, in the case of CVA, GERD, chronic bronchitis, eosinophilic bronchitis, nonspecific chronic cough, and unexplained chronic cough, more than 50% of the studies involved adults (S4 Appendix).

### EATM treatment

**Fig 3:**
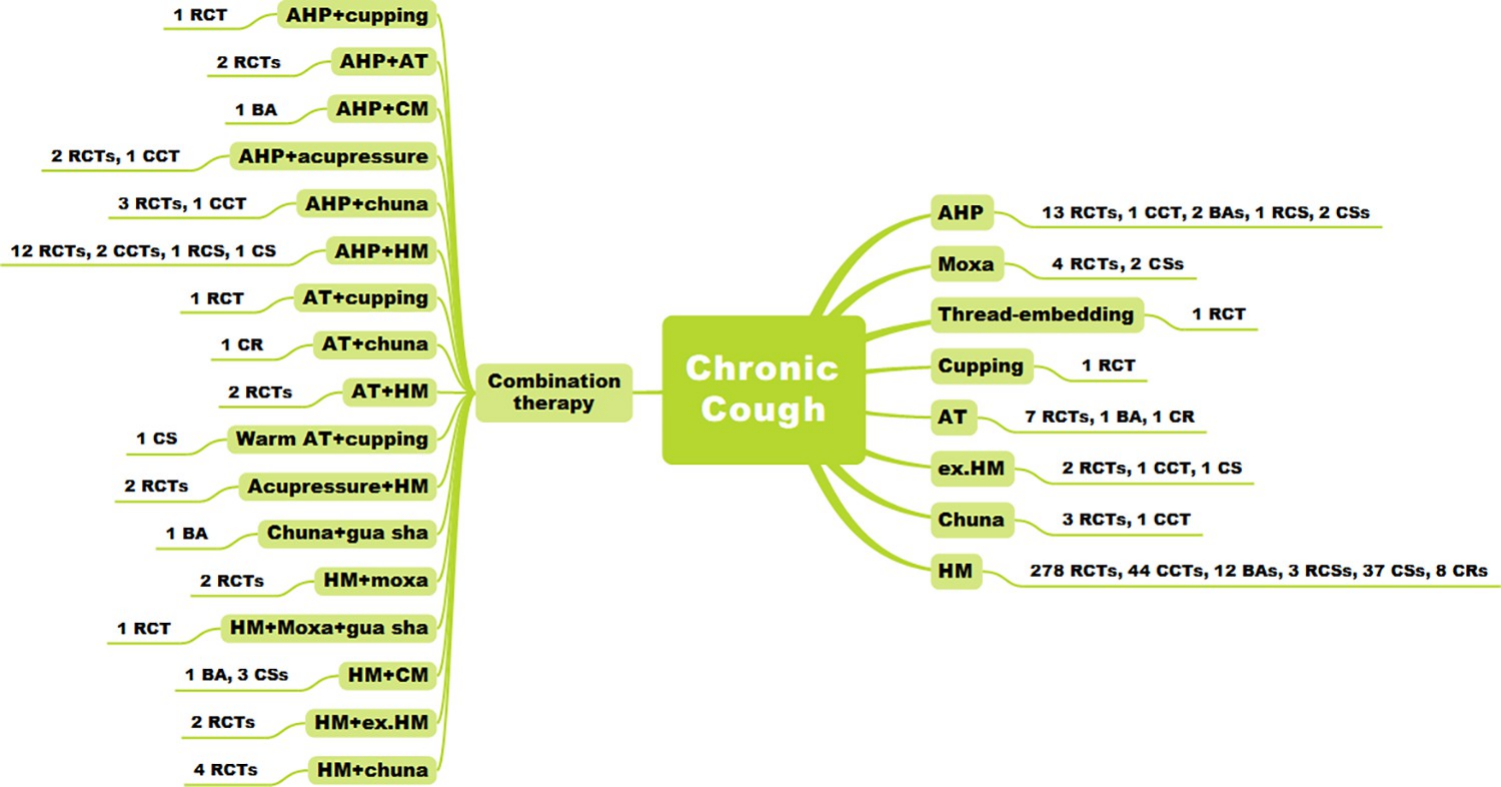
Research status according to the EATM treatment. Abbreviations. AHP, acupoint herbal patching; AT, acupuncture; BA, before-after study; CCT, non-randomized controlled clinical trial; CM, conventional medication; CR, case report; CS, case series; ex.HM, external application of herbal medicine; HM, herbal medicine; Moxa, moxibustion; RCS, retrospective cohort study; RCT, randomized controlled clinical trial.

**Fig 4:**
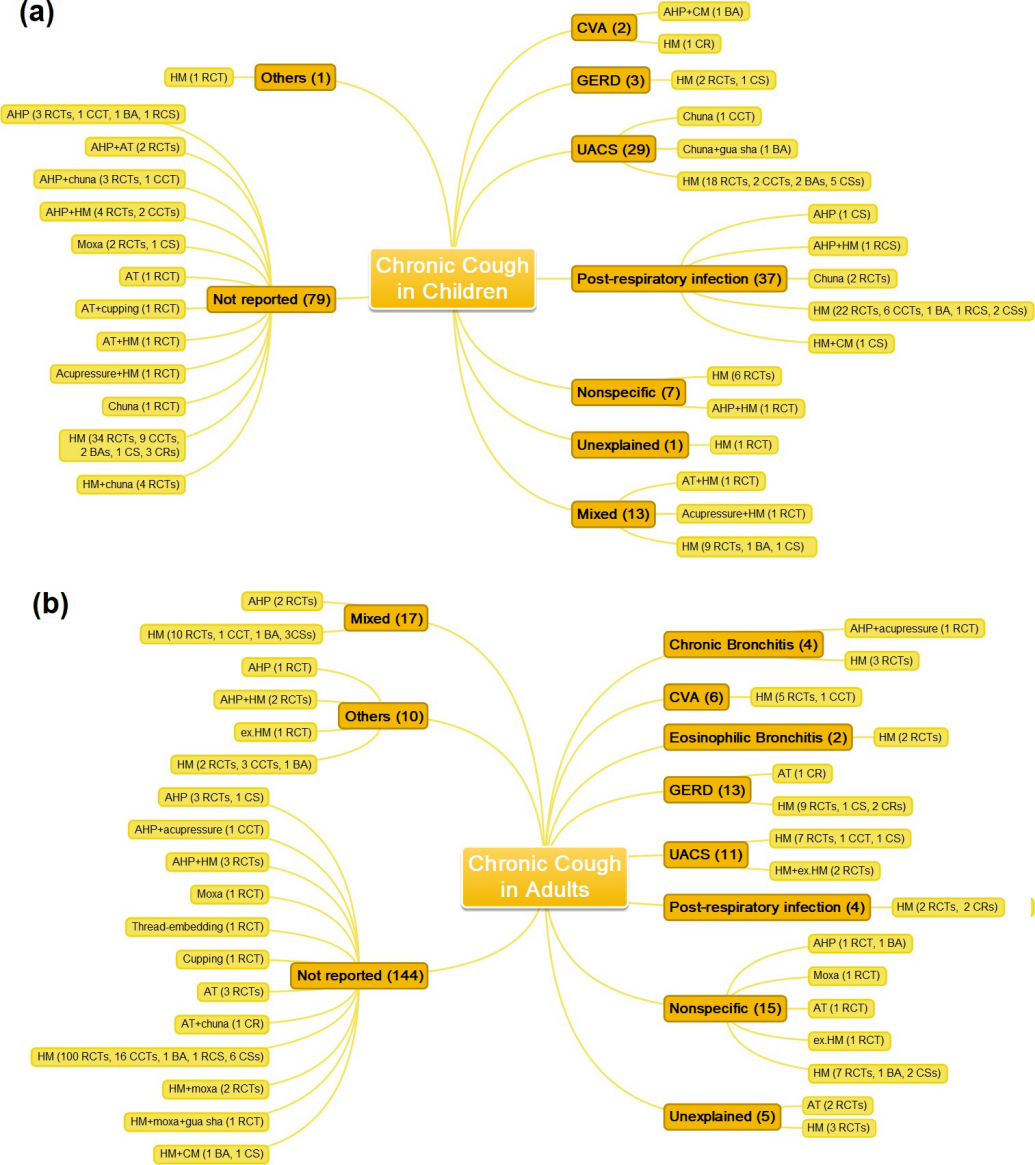
Research status according to the cause of cough and EATM treatment in (a) children and (b) adults. Abbreviations. AHP, acupoint herbal patching; AT, acupuncture; BA, before-after study; CCT, non-randomized controlled clinical trial; CM, conventional medication; CR, case report; CS, case series; CVA, cough variant asthma; ex.HM, external application of herbal medicine; GERD, gastroesophageal reflux disease; HM, herbal medicine; Moxa, moxibustion; RCS, retrospective cohort study; RCT, randomized controlled clinical trial; UACS, upper airway cough syndrome.

Among the EATM treatments used in the included studies, herbal medicine alone was used in most of the studies (382 studies, 80.6%). Acupoint herbal patching (AHP) (19 studies, 4.0%), AHP plus herbal medicine (16 studies, 3.4%), manual acupuncture (nine studies, 1.9%), and moxibustion (six studies, 1.3%) were also frequently used (Fig 3). The current status of EATM clinical studies according to the cause of cough in children and adults is presented in Figs 4(A) and 4(B). After excluding 10 studies that did not report the treatment period and 41 studies that indicated only the range, the average EATM treatment period in the remaining 423 studies was 19.1 ± 13.6 days (range, 3~90 days). There were 399 studies with control groups (343 RCTs, 51 CCTs, and five retrospective cohort studies). Conventional medication alone was used in most controlled studies (324 studies, 81.2%), and herbal medicine was also used frequently in 49 (12.3%) studies. The number of studies using sham acupuncture and placebo drug was one each (0.3%).

### Outcome measures and results

Most studies (447 studies, 94.3%) reported the TER based on cough symptoms, followed by cough severity in 255 (53.8%) studies, adverse events in 164 (34.6%) studies, blood biomarkers in 58 (12.2%) studies, cough-related quality of life in 52 (11.0%) studies, pulmonary function test indices in 31 (6.5%) studies, and cough frequency in nine (1.9%) studies.

The results were divided into three categories (“Positive outcomes”, “Not different from a control”, and “Inconsistent”). Based on the results of cough severity, cough frequency, cough-related quality of life, TER, and blood biomarkers, more than 90% of studies determined that EATM treatment showed positive outcomes for chronic cough. Based on pulmonary function test indices, EATM treatment was statistically significant in 87.1% of the studies, and 9.7% of the studies showed no significant difference when compared with a control group (Table 2).

**Table 2:** Outcomes and results of the included studies.

Outcomes and results	N	%
**Cough severity**		
Positive outcomes	242	94.5%
Not different from a control	11	4.3%
Inconsistent	3	1.2%
Total	256	100.0%
**Cough frequency**		
Positive outcomes	9	100.0%
Total	9	100.0%
**Cough-related quality of life**		
Positive outcomes	49	92.5%
Not different from a control	4	7.5%
Total	53	100.0%
**Total effective rate**		
Positive outcomes	426	95.3%
Not different from a control	21	4.7%
Total	447	100.0%
**Blood biomarker**		
Positive outcomes	55	94.8%
Not different from a control	2	3.4%
Inconsistent	1	1.7%
Total	58	100.0%
**Pulmonary function test indices**		
Positive outcomes	27	87.1%
Not different from a control	3	9.7%
Inconsistent	1	3.2%
Total	31	100.0%
**Adverse events**		
Reported	164	34.6%

## Discussion

Through a comprehensive study search, a total of 474 clinical studies were included. A review of the general characteristics of the included studies revealed that a number of relevant studies were conducted in the past 10 years (2012~2022), and interventional studies showed an increasing trend. These results might imply that the current trends have shifted to evaluation of evidence-based efficacy/effectiveness of specific EATM interventions rather than observational studies on its use and impact, due to the growing need for alternative treatments for chronic cough. Most studies did not report the cause of chronic cough. All of these studies were conducted in China, published in Chinese, and only described patients with chronic cough. In addition, no information revealed the cause of cough, including causative conditions and specific/nonspecific coughs. In conventional medicine, treatment strategies differ depending on the cause of cough. Although EATM has its own diagnosis system and a pattern identification questionnaire for chronic cough [25], it is necessary to consider not only the pattern but also the cause of cough to determine the appropriate therapeutic approach. Therefore, in future studies, in the case of a specific cough, the cause should be stated, and in the case of a nonspecific cough, this should be indicated clearly. Additionally, to inform more readers around the world of the clinical effects of EATM, future related clinical studies should be published in international journals in English.

Among the EATM treatments, herbal medicine alone was the most often utilized, but EATM combination therapy was also frequently used, which may be attributed to the use of various EATM treatment combinations in clinical settings. EATM treatment showed positive outcomes with the main outcome measures. In controlled studies, conventional medication was mostly used as the control, and EATM treatment was significantly more effective than the control, showing no difference from the control in less than 10% of the studies. In particular, herbal medicine is characterized as involving multiple components and multiple targets [26]. Therefore, unlike conventional medication, which is used for treatment according to the cause, herbal medicine is promising not only for cough with a cause but also for cough whose cause is not specified or cannot be explained. In general, there are many causes of chronic cough, and it is a condition that can appear as a symptom with a combination of various causes [27]. It is not easy to identify the main cause of cough [5]; thus, empirical treatment is performed [28, 29]. In this case, herbal medicine having multi-components multi-targets characteristics might be helpful. Accordingly, studies evaluating the effect of EATM treatment on nonspecific or unexplained chronic cough should be conducted in the future. For this purpose, according to the definition of nonspecific or unexplained chronic cough [10, 11], history taking, physical examination, radiography, and spirometry should be performed to determine the cause of cough, and patients whose cause is unknown or who do not improve after appropriate therapeutic trials should be recruited. In addition, nonpharmacological interventions such as AHP, manual acupuncture, and moxibustion were also frequently used in the included studies. Due to the nature of nonpharmacological therapy, it is a safe treatment if performed by an experienced practitioner [30, 31], and its clinical effectiveness and safety for cough have been reported in previous studies [32–34].

As most of the studies were conducted in China, the TER based on chronic cough symptoms was described in most of the included Chinese studies, and a significant number of studies declared only TER results without other indicators. To the best of our knowledge, a core outcome set for chronic cough has not been developed; however, several clinical practice guidelines have recommended the use of standardized cough assessment tools [35–37]. In addition, a cough-specific health-related quality of life questionnaire is considered to be the most comprehensive evaluation of the impact of cough on patients, and its use in cough-related clinical studies has been recommended [35, 37]. Therefore, to objectively and accurately assess the effect of EATM treatment, standardized and objective evaluation tools such as the CQLQ, LCQ, and Chronic Cough Impact Questionnaire (CCIQ) should be used in future clinical studies [38–40]. In addition, the features of EATM, such as pattern identification, should be reflected when assessing the effect of EATM treatment, and it is necessary to develop a core outcome set for chronic cough.

Based on the findings, we proposed the PICOs for a systematic review of EATM treatment of chronic cough as follows. First, for the population, various studies have been conducted on post-respiratory infection and UACS in children and on nonspecific chronic cough and GERD in adults. Therefore, it will be possible to analyze the effect size of EATM on chronic cough associated with these causes in children and adults. If an inclusive population is selected for patients with chronic cough broadly, it is necessary to analyze subgroups according to age (children and adults) and diseases that cause cough. Second, for the type of intervention in systematic reviews, the effects of herbal medicine or acupuncture-related therapy (acupuncture, moxibustion, and AHP) on chronic cough regardless of its cause can be summarized. Third, for the comparators, conventional medication is usually used as a control group and there are limited studies using a placebo control (sham acupuncture and placebo drug). Therefore, clinical questions that evaluate efficacy by setting the comparator only as a placebo control are not appropriate. Fourth, for outcome measures, they can be established by referring to the frequently reported outcome measures identified in this review (cough severity, cough-related quality of life, and TER), and outcomes recommended in clinical practice guidelines [35–37].

This study has some limitations. We established the search strategy including free words and index words corresponding to “chronic cough” and did not include search terms such as UACS, CVA, and GERD corresponding to the cause of cough. Therefore, there might be related studies that have not been analyzed in this review. In addition, to achieve the purpose of the scoping review, which rapidly maps the range and number of available studies, we attempted to present comprehensive evidence by conducting searches using various databases with increased accuracy.

Nevertheless, this is the first study to examine the clinical research status of EATM treatment for chronic cough using a comprehensive and rigorous methodology. We attempted to minimize reporting bias by registering the research protocol in a registry, and through mapping (a key element of scoping reviews), the scope of the study could be visualized and confirmed at a glance. Based on the findings, systematic reviews that summarize the effect size and safety of various EATM treatments and evaluate the methodological quality of individual studies and the quality of evidence for the combined effect estimate should be conducted, which may aid the clinical decision-making of clinicians, patients, and researchers. In the future, relevant clinical studies should be conducted not only in China but also in countries such as Korea, Japan, and Taiwan. Furthermore, clinical studies should be conducted on the cause of cough and EATM treatment, which lacked clinical evidence as identified in this review.

Clinical research on EATM for chronic cough illustrated that the cause of cough was not presented in most studies, and the most common cause was upper airway cough syndrome and post-respiratory infection. Herbal medicine alone was the most utilized treatment, and conventional medication was frequently used as a control. For outcomes, TER and cough severity were often assessed. In future EATM studies, it is necessary to specify the cause of chronic cough or to state that the study was targeting nonspecific chronic cough. In addition, high-quality studies assessing the efficacy of EATM with placebo control should be conducted, using validated evaluation tools.

## Supporting information

S1 Appendix Search strategies used in each database and the results. (DOCX)

S2 Appendix Excluded studies after a full-text review. (DOCX)

S3 Appendix References of the included studies. (DOCX)

S4 Appendix Cause of cough in the included studies according to age. (DOCX)
