# Supplementary material for: Research status of east Asian traditional medicine treatment for chronic cough: A scoping review
Source: PLoS One. 2024 Feb 8;19(2):e0296898. doi: 10.1371/journal.pone.0296898 (PMC10852285; doi:10.1371/journal.pone.0296898)
Supplement: S1 Appendix — (DOCX) [file pone.0296898.s001.docx]

**S1 Appendix. Search strategies used in each database and the results**

**Medline via PubMed**

|  | Searches | Results |
| --- | --- | --- |
| #1 | chronic[TIAB] AND cough[TIAB] | 10630 |
| #2 | “Herbal Medicine”[MH] OR “Plants, Medicinal”[MH] OR “Drugs, Chinese Herbal”[MH] OR “Medicine, Chinese Traditional”[MH] OR “Medicine, Kampo”[MH] OR “Medicine, Korean Traditional”[MH] OR “traditional Korean medicine”[TIAB] OR “traditional Chinese medicine”[TIAB] OR “traditional oriental medicine”[TIAB] OR “Kampo medicine”[TIAB] OR herb*[TIAB] OR decoction*[TIAB] OR botanic*[TIAB] OR “Chinese patent medicine”[TIAB] OR Acupuncture[MH] OR “Acupuncture Therapy”[MH] OR Auriculotherapy[MH] OR “Acupuncture, Ear”[MH] OR Acupressure[MH] OR Electroacupuncture[MH] OR “Acupuncture Points”[MH] OR acupuncture[TIAB] OR acupressure[TIAB] OR acupoint*[TIAB] OR “ear acupuncture”[TIAB] OR electroacupuncture[TIAB] OR electro‐acupuncture[TIAB] OR pharmacopuncture[TIAB] OR pharmaco-acupuncture[TIAB] OR “Bee Venoms”[MH] OR “bee venom*”[TIAB] OR “acupoint injection”[TIAB] OR Moxibustion[MH] OR moxibustion[TIAB] OR moxa[TIAB] OR “warm needling”[TIAB] OR “Tai Ji”[MH] OR “Tai Chi”[TIAB] OR “T’ai Chi”[TIAB] OR Taiji[TIAB] OR “Tai ji”[TIAB] OR taijiquan[TIAB] OR “Tai Chih”[TIAB] OR “Cupping Therapy”[MH] OR “cupping therapy”[TIAB] OR Qigong[MH] OR “qi gong”[TIAB] OR qigong[TIAB] OR Massage[MH] OR massage[TIAB] OR tuina[TIAB] OR chuna[TIAB] OR manipulat*[TIAB] OR “manual therapy”[TIAB] OR Chiropractic[TIAB] OR “Musculoskeletal Manipulations”[MH] OR “Manipulation, Spinal”[MH] OR “Manipulation, Chiropractic”[MH] | 527015 |
| #3 | #1 AND #2 | **218** |

**EMBASE via Elsevier**

|  | Searches | Results |
| --- | --- | --- |
| #1 | ‘chronic cough’/exp OR (chronic:ab,ti AND cough:ab,ti) | 21922 |
| #2 | ‘medicinal plant’/exp OR ‘medicinal plant’:ab,ti OR ‘plant medicinal product’/exp OR ‘plant medicinal product’:ab,ti OR ‘herbaceous agent’/exp OR ‘herbaceous agent’:ab,ti OR ‘chinese medicine’/exp OR ‘chinese medicine’:ab,ti OR ‘kampo medicine’/exp OR ‘kampo medicine’:ab,ti OR ‘kampo medicine (drug)’/exp OR ‘kampo medicine (drug)’:ab,ti OR ‘korean medicine’/exp OR ‘korean medicine’:ab,ti OR ‘herbal medicine’/exp OR ‘herbal medicine’:ab,ti OR ‘oriental medicine’/exp OR ‘oriental medicine’:ab,ti OR herb/exp OR herb:ab,ti OR decoction:ab,ti OR botanic:ab,ti OR ‘chinese patent medicine’:ab,ti OR acupuncture/exp OR acupuncture:ab,ti OR ‘acupuncture therapy’:ab,ti OR ‘auricular acupuncture’/exp OR ‘auricular acupuncture’:ab,ti OR ‘ear acupuncture’:ab,ti OR auriculotherapy:ab,ti OR acupressure/exp OR acupressure:ab,ti OR electroacupuncture/exp OR electroacupuncture:ab,ti OR ‘electro-acupuncture’:ab,ti OR ‘acupuncture point’/exp OR ‘acupuncture point’:ab,ti OR acupoint:ab,ti OR pharmacopuncture/exp OR pharmacopuncture:ab,ti OR ‘pharmaco-acupuncture’:ab,ti OR 'bee venom'/exp OR 'bee venom':ab,ti OR 'acupoint injection':ab,ti OR moxibustion/exp OR moxibustion:ab,ti OR moxa:ab,ti OR 'warm needling':ab,ti OR ‘Tai Chi’/exp OR ‘Tai Chi’:ab,ti OR Taiji:ab,ti OR ‘Tai ji’:ab,ti OR taijiquan:ab,ti OR ‘Tai Chih’:ab,ti OR 'cupping therapy'/exp OR 'cupping therapy':ab,ti OR qigong/exp OR qigong:ab,ti OR 'qi gong':ab,ti OR massage/exp OR massage:ab,ti OR tuina/exp OR tuina:ab,ti OR chuna:ab,ti OR 'manipulative medicine'/exp OR 'manipulative medicine':ab,ti OR 'manual therapy':ab,ti OR chiropractic/exp OR chiropractic:ab,ti OR 'musculoskeletal manipulation'/exp OR 'musculoskeletal manipulation':ab,ti OR 'spine manipulation'/exp OR 'spine manipulation':ab,ti OR 'chiropractic manipulation'/exp OR 'chiropractic manipulation':ab,ti | 1979138 |
| #3 | #1 AND #2 | **1312** |

**CENTRAL**

|  | Searches | Results |
| --- | --- | --- |
| #1 | (chronic AND cough):ti.ab.kw | 2380 |
| #2 | MeSH descriptor: [Herbal Medicine] explode all trees | 68 |
| #3 | MeSH descriptor: [Plants, Medicinal] explode all trees | 953 |
| #4 | MeSH descriptor: [Drugs, Chinese Herbal] explode all trees | 3806 |
| #5 | MeSH descriptor: [Medicine, Chinese Traditional] explode all trees | 1276 |
| #6 | MeSH descriptor: [Medicine, Kampo] explode all trees | 48 |
| #7 | MeSH descriptor: [Medicine, Korean Traditional] explode all trees | 34 |
| #8 | MeSH descriptor: [Acupuncture] explode all trees | 164 |
| #9 | MeSH descriptor: [Acupuncture Therapy] explode all trees | 5326 |
| #10 | MeSH descriptor: [Auriculotherapy] explode all trees | 260 |
| #11 | MeSH descriptor: [Acupuncture, Ear] explode all trees | 221 |
| #12 | MeSH descriptor: [Acupressure] explode all trees | 427 |
| #13 | MeSH descriptor: [Electroacupuncture] explode all trees | 889 |
| #14 | MeSH descriptor: [Acupuncture Points] explode all trees | 2261 |
| #15 | MeSH descriptor: [Bee Venoms] explode all trees | 45 |
| #16 | MeSH descriptor: [Moxibustion] explode all trees | 522 |
| #17 | MeSH descriptor: [Tai Ji] explode all trees | 408 |
| #18 | MeSH descriptor: [Cupping Therapy] explode all trees | 17 |
| #19 | MeSH descriptor: [Qigong] explode all trees | 97 |
| #20 | MeSH descriptor: [Massage] explode all trees | 1295 |
| #21 | MeSH descriptor: [Musculoskeletal Manipulations] explode all trees | 3360 |
| #22 | MeSH descriptor: [Manipulation, Spinal] explode all trees | 431 |
| #23 | MeSH descriptor: [Manipulation, Chiropractic] explode all trees | 139 |
| #24 | (“traditional Korean medicine” OR “traditional Chinese medicine” OR “traditional oriental medicine” OR “Kampo medicine” OR herb* OR decoction* OR botanic* OR “Chinese patent medicine” OR acupuncture OR acupressure OR acupoint* OR “ear acupuncture” OR electroacupuncture OR pharmacopuncture OR pharmacoacupuncture OR “bee venom*” OR “acupoint injection” OR moxibustion OR moxa OR “warm needling” OR “Tai Chi” OR “T’ai Chi” OR Taiji OR “Tai ji” OR taijiquan OR “Tai Chih” OR “cupping therapy” OR “qi gong” OR qigong OR massage OR tuina OR chuna OR manipulat* OR “manual therapy” OR Chiropractic):ti,ab,kw | 60263 |
| #25 | #2 OR #3 OR #4 OR #5 OR #6 OR #7 OR #8 OR #9 OR #10 OR #11 OR #12 OR #13 OR #14 OR #15 OR #16 OR #17 OR #18 OR #19 OR #20 OR #21 OR #22 OR #23 OR #24 | 61376 |
| #26 | #1 AND #25 | **98** |

**OASIS**

|  | Searches | Results |
| --- | --- | --- |
| #1 | (만성기침) (한약\|약초\|본초\|침\|혈위\|지압\|봉독\|뜸\|태극권\|부항\|기공\|추나\|안마) | **1** |

**CNKI**

|  | Searches | Results |
| --- | --- | --- |
| #1 | (SU='慢性咳嗽'+'长期咳嗽') AND (SU='中药'+'中医药'+'本草'+'汤'+'丸'+'散'+'颗粒'+'胶囊'+’针’+'指压'+'按压'+'耳压'+’穴位’+’蜂毒‘+’蜂疗‘+’穴位注射‘+’按摩'+'推拿'+'太极拳'+'气功'+'灸'+'火罐'+'拔罐') | **1260** |

**CiNii**

|  | Searches | Results |
| --- | --- | --- |
| #1 | (**慢性咳嗽 OR 慢性咳 OR 慢性的な咳**) AND (ハーブ OR 漢方薬 OR 中药 OR 本草 OR 鍼 OR 指圧 OR しあつ OR 按圧 OR 耳圧 OR 穴位 OR はちどく OR 蜂毒 OR 蜂疗 OR 穴位注射 OR マッサージ OR カイロプラクティック OR 按摩 OR 推拿 OR 太極拳 OR 気功 OR 灸 OR 吸角 OR 拔罐 OR カッピング) | **40** |
