# Supplementary material for: Research status of east Asian traditional medicine treatment for chronic cough: A scoping review
Source: PLoS One. 2024 Feb 8;19(2):e0296898. doi: 10.1371/journal.pone.0296898 (PMC10852285; doi:10.1371/journal.pone.0296898)
Supplement: S2 Appendix — (DOCX) [file pone.0296898.s002.docx]

**S2 Appendix. Excluded studies after full-text review**

**1) Not about chronic cough (n = 95)**

1. 高陆地, *三联疗法治疗慢性咳嗽48例.* 实用中医药杂志, 2012. **28**(07): p. 575-576.

2. 郭建明, *浅议中药治疗慢性咳嗽.* 内蒙古中医药, 2015. **34**(06): p. 17-18.

3. 郭召平, 马淑颖, and 朱生樑. *丁香降气汤治疗胃食管反流性咳嗽41例临床观察*. in *中华中医药学会第二十二届全国脾胃病学术交流会暨2010年脾胃病诊疗新进展学习班*. 2010. 中国江西井冈山.

4. 年云娜, *银翘散加减汤剂治疗小儿慢性咳嗽的临床体会.* 中国城乡企业卫生, 2014. **29**(01): p. 167-168.

5. 唐晓文, et al., *无痛针灸配合中药水罐治疗慢性咳嗽的临床疗效.* 临床检验杂志(电子版), 2016. **5**(01): p. 25-28.

6. 童玉琴, *自拟理中止嗽散治疗慢性咳嗽80例——附西药治疗80例对照.* 浙江中医杂志, 2004(02): p. 19.

7. 李宏伟, 温树辉, and 张楠, *止嗽散配合超短波疗法治疗感冒后慢性咳嗽临床观察.* 中医药临床杂志, 2014. **26**(01): p. 30-31.

8. 李芳, *补气活血法联合艾灸治疗儿童慢性咳嗽89例.* 浙江中医杂志, 2016. **51**(02): p. 111.

9. 李小慧, 张素卿, and 李巧兰, *沙参麦冬汤加减治疗老年慢性咳嗽体会.* 实用中医药杂志, 2013. **29**(04): p. 287.

10. 李崇进, *壮医龙盘止咳方治疗小儿咳嗽的中医证效研究*. 2019, 广西中医药大学.

11. 李雅莉, *宣降止咳汤治疗小儿慢性咳嗽的临床疗效观察.* 航空航天医学杂志, 2011. **22**(07): p. 892-893.

12. 李婷婷, *益气通窍止咳汤治疗上气道咳嗽综合征（肺脾气虚风痰阻窍型）临床分析*. 2010, 湖北中医药大学.

13. 李天庆, *含有麦门冬汤等多种药物并用疗法治疗感冒后综合征的慢性咳嗽.* 国外医学(中医中药分册), 1998(02): p. 31.

14. 李海宁, *止咳散加味治疗外感后慢性咳嗽46例.* 实用中医内科杂志, 2006(02): p. 189.

15. 林芳, *拔罐加TDP治疗慢性咳嗽31例.* 福建中医药, 2005(01): p. 28-29.

16. 林锡丰, *止咳散加味治疗外感后慢性咳嗽55例的临床疗效分析.* 中医临床研究, 2015. **7**(01): p. 104-105.

17. 李达仁 and 陈碧云, *利咽平喘活血方治疗慢性咳嗽69例.* 上海中医药杂志, 2001(07): p. 27.

18. 苗良, *参苓汤治疗慢性咳嗽64例.* 实用中医内科杂志, 2008(09): p. 19.

19. 常南军 and 王慧敏, *加味止嗽散治疗慢性咳嗽60例.* 河南中医, 2014. **34**(07): p. 1346-1347.

20. 盛萍, *中药治疗慢性咳嗽30例临床体会.* 青海医药杂志, 2010. **40**(05): p. 71-73.

21. 邵丽, 梁尤娟, and 谢进, *沙参麦冬汤合芍药甘草汤对肺炎支原体感染后小儿慢性咳嗽的治疗效果观察.* 临床合理用药杂志, 2017. **10**(17): p. 70-71.

22. 余成勇, *苓甘五味姜辛汤合止嗽散加减治疗慢性咳嗽临床观察.* 新中医, 2017. **49**(07): p. 44-46.

23. 王佳芳, *清金化痰冲剂治疗小儿痰热咳嗽的临床观察*. 2011, 黑龙江中医药大学.

24. 王黎芸, *养阴止嗽汤治疗慢性咳嗽56例.* 云南中医中药杂志, 1999(04): p. 29.

25. 王寅平, *小五味子汤联合布地奈德治疗风邪伏肺型呼吸道感染后慢性咳嗽的临床观察.* 中国民间疗法, 2022. **30**(02): p. 84-86.

26. 王海秀 and 杨宝, *蒿芩清胆汤治疗慢性咳嗽验案一例.* 中国药物与临床, 2007(08): p. 630.

27. 王壮, *清肺化痰汤治疗小儿咳嗽痰热壅肺型的临床研究*. 2019, 山西中医药大学.

28. 王开新, *参百止咳汤治疗慢性咳嗽130例.* 实用中医药杂志, 1999(01): p. 22.

29. 熊杰, 王成艳, and 年云娜. *中药治疗慢性咳嗽的体会*. in *第五次全国中西医结合呼吸病学术交流大会*. 2000. 中国南昌.

30. 熊霖, et al., *中医内外结合治疗小儿感染后咳嗽的临床研究.* 中国中医基础医学杂志, 2021. **27**(10): p. 1619-1621+1679.

31. 熊新军, *清肺止咳口服液治疗慢性支气管炎急性发作（风邪化热壅肺型咳嗽）的临床研究*. 2007, 湖北中医学院.

32. 尹双红, *自拟反咳汤治疗咳嗽的临床观察.* 中国民间疗法, 2020. **28**(05): p. 35-36.

33. 尹鸣, *中药浓煎剂小儿止咳方在慢性咳嗽中的应用体会.* 中西医结合心血管病电子杂志, 2015. **3**(06): p. 77-78.

34. 任文辉, *益气化痰汤治疗慢性咳嗽68例.* 河南中医, 2006(07): p. 40-41.

35. 周敏 and 裴秋兰, *止嗽散治疗慢性咳嗽临床辨证应用体会.* 亚太传统医药, 2020. **16**(01): p. 104-105.

36. 周桦, *小青龙汤加减治疗鼻后滴漏综合征的临床分析.* 世界最新医学信息文摘, 2016. **16**(82): p. 197+18.

37. 朱砚萍, *治咳方治疗慢性咳嗽110例.* 上海中医药杂志, 2003(05): p. 35-36.

38. 周继朴, *王莒生教授学术思想与临床经验总结及从五脏论治慢性咳嗽临床经验研究*. 2011, 北京中医药大学.

39. 周贺芙, *慢性咳嗽从虚寒论治临床研究*. 2007, 长春中医药大学.

40. 周贺芙 and 杨海淼, *从虚寒论治慢性咳嗽临床观察80例.* 吉林中医药, 2007(04): p. 22-23.

41. 秦泗明, *温肺止咳汤治疗咳嗽38例.* 陕西中医, 1997(04): p. 161.

42. 秦国琦, *中西医结合治疗咳嗽变异型哮喘的临床观察*. 2010, 黑龙江中医药大学.

43. 蔡安琼, *养阴清肺汤改善阴虚肺热证小儿肺炎恢复期慢性咳嗽的临床效果.* 中外医学研究, 2021. **19**(17): p. 51-53.

44. 何胜恬, *祛风通窍汤治疗鼻后滴漏型慢性咳嗽112例.* 浙江中医杂志, 2007(07): p. 410.

45. 叶秋婷, *止嗽散化裁治疗顽咳60例.* 实用中西医结合临床, 2004(01): p. 52-53.

46. 邢海玲, *麻杏石甘汤加减治疗呼吸道感染后慢性咳嗽的临床效果观察探讨.* 医学食疗与健康, 2020. **18**(05): p. 44+46.

47. 胡浩亮 and 罗桂平, *止嗽散治疗小儿感染后咳嗽临床研究.* 光明中医, 2022. **37**(03): p. 451-453.

48. 胡妮娜, *半夏厚朴汤联合常规西药治疗慢性咳嗽的有效性及复发率.* 现代养生, 2021. **21**(10): p. 44-46.

49. 胡绍贵, 李红杰, and 彭海平, *止嗽散加味治疗慢性咳嗽229例.* 实用中医内科杂志, 2005(06): p. 565.

50. 农志新, *麻黄附子细辛汤加减治疗慢性咳嗽50例.* 福建中医药, 2007(03): p. 24.

51. 刘方毅, *培土生金法联合穴位贴敷曼吉磁贴治疗小儿慢性咳嗽的疗效观察.* 护理实践与研究, 2015. **12**(08): p. 145-146.

52. 刘小平, *六君子汤治疗慢性咳嗽60例报道.* 甘肃中医, 2001(06): p. 15-16.

53. 刘颖, *中药颗粒治疗慢性咳嗽的临床效果分析.* 内蒙古中医药, 2016. **35**(14): p. 45.

54. 吴美美, *牛子利咽汤治疗风邪犯肺型喉源性咳嗽的临床疗效观察*. 2015, 山东中医药大学.

55. 吴启富, 赵将, and 吴江华, *疏风解毒胶囊联合常规治疗上气道咳嗽综合征的疗效观察.* 中国药师, 2018. **21**(05): p. 856-858.

56. 吴静南 and 唐国良, *三拗汤加味治疗慢性咳嗽疗效观察.* 新中医, 2015. **47**(09): p. 56.

57. 孙靖峰, *芪蝉止咳汤联合复方磷酸可待因治疗慢性咳嗽31例.* 河北中医, 2012. **34**(12): p. 1823-1824.

58. 张雷英, *止嗽散合黛蛤散治疗外感后慢性咳嗽肝火犯肺证40例.* 中国中医药科技, 2018. **25**(06): p. 896-898.

59. 张雯, *益气活血肃肺汤治疗小儿慢性咳嗽（肺脾气虚型）的临床研究*. 2004, 山东中医药大学.

60. 张雯, 陈丽萍, and 王伟学, *益气活血肃肺汤治疗小儿慢性咳嗽(肺脾气虚型)的临床研究.* 中华中医药学刊, 2008(02): p. 447-448.

61. 张茵, *滋阴止咳汤治疗小儿慢性咳嗽的临床效果分析.* 中国医药指南, 2019. **17**(21): p. 174-175.

62. 张婧 and 齐迅捷, *杏苏散加减联合艾灸治疗慢性咳嗽41例疗效观察.* 中国民族民间医药, 2017. **26**(01): p. 102+106.

63. 张俊红, *麻杏薏甘汤合止嗽散治疗慢性咳嗽54例.* 中医研究, 2011. **24**(03): p. 19-20.

64. 张志娜, 曹洪全, and 孟要武, *清金化痰汤配合针刺四缝穴对慢性咳嗽的疗效观察.* 中医临床研究, 2020. **12**(20): p. 25-26.

65. 张平, 王海东, and 董兰芬, *茯苓白术汤治疗慢性咳嗽58例.* 中国民间疗法, 2003(03): p. 47-48.

66. 时高波, *射干麻黄汤治疗寒性咳嗽的临床观察*. 2011, 广州中医药大学.

67. 杨梅玉, *麻杏石甘汤治疗呼吸道感染后慢性咳嗽的临床效果.* 中国当代医药, 2022. **29**(08): p. 141-143.

68. 杨文华, *加味玉屏风散治疗慢性咳嗽93例.* 内蒙古中医药, 2010. **29**(01): p. 75.

69. 杨玉霞, 刘丽华, and 张风敏, *针刺背俞穴配合穴位埋线治疗慢性咳嗽49例疗效观察.* 河北中医, 2012. **34**(07): p. 1039-1040.

70. 杨超, *二陈汤加减治疗慢性咳嗽120例临床观察.* 临床医药文献电子杂志, 2014. **1**(12): p. 2082-2083.

71. 杨伟岸, *桑柴汤加减治疗慢性咳嗽33例.* 实用中医药杂志, 1999(04): p. 39.

72. 杨凤仙 and 黄振炎, *理中丸治疗慢性咳嗽86例.* 福建中医药, 2004(04): p. 31-32.

73. 罗森, *止嗽散加减治疗慢性咳嗽37例.* 临床医药实践, 2009. **18**(26): p. 2058-2059.

74. 赖伟萍, *舒咽止咳汤治疗咽源性咳嗽的临床观察*. 2019, 浙江中医药大学.

75. 赵宪平 and 花亚历, *孟鲁司特钠、布地奈德联合小青龙汤加减治疗嗜酸粒细胞性支气管炎的临床观察.* 中国民间疗法, 2019. **27**(15): p. 73-74.

76. 赵颖, *中药浓煎剂小儿止咳方治疗慢性咳嗽效果分析.* 中国中医药现代远程教育, 2016. **14**(11): p. 97-98.

77. 邓成秀, *加味二陈汤治疗小儿痰湿蕴肺型慢性咳嗽的临床分析.* 中医临床研究, 2019. **11**(36): p. 33.

78. 邹怡 and 关玲, *耳穴贴压配合拔罐治疗小儿慢性咳嗽.* 中国民间疗法, 2009. **17**(09): p. 24.

79. 郑志勇, *加味泻白散治疗小儿慢性咳嗽（肺热阴虚型）的临床研究*. 2006, 成都中医药大学.

80. 陆岗, *一贯煎加味治疗慢性咳嗽（肝肾阴虚证）的机理探讨和临床研究*. 2012, 长春中医药大学.

81. 陈冬梅 and 王新佩, *穴位贴敷治疗慢性咳嗽56例.* 中医外治杂志, 2015. **24**(04): p. 13.

82. 陈安, *益气补肺汤辅助治疗慢性咳嗽的疗效分析.* 系统医学, 2021. **6**(11): p. 156-158.

83. 陈坚志, *养阴通窍利咽汤治疗小儿鼻液后滴综合征48例.* 湖南中医杂志, 2005(06): p. 56.

84. 陈瑶, *中医治疗慢性咳嗽临床效果观察.* 内蒙古中医药, 2017. **36**(18): p. 20-21.

85. 马恩明. *慢性咳嗽19例临床初步分析(摘要)*. in *全国中西医结合耳鼻咽喉科学术会议*. 2005. 中国重庆.

86. 马志杰, *保肺滋肾汤联合西药治疗慢性咳嗽46例.* 中医研究, 2015. **28**(11): p. 33-34.

87. 马进, *止嗽降气汤治疗慢性咳嗽120例的临床观察.* 内蒙古中医药, 2013. **32**(20): p. 12.

88. 黄河清 and 韩林华, *采用降气为主、肝脾同治法治疗慢性咳嗽84例疗效分析.* 中国医药指南, 2008. **6**(24): p. 174-176.

89. 黄进, 刘八一, and 邓小敏, *半夏泻心汤化裁治疗慢性咳嗽.* 广西中医学院学报, 2005(02): p. 44-45.

90. Fujimori, K., K. Sakurai, and A. Yoshizumi, *A case of postinfectious chronic cough improved with bakumondo-to not with antihistamine and dextromethorphan hydrobromide.* Arerugī = [Allergy], 1995. **44**(12): p. 1418-1421.

91. Kim, E. and H. Jo, *Treatment of Chronic Cough in an Upper Airway Cough Syndrome (UACS)-Suspected Patient with Local Acupuncture Points Stimulation and Application of Topical Herbal Mixed Heating Cream : A Case Report.* 대한한방내과학회지, 2019. **40**(3): p. 557-565.

92. Qiao, M., et al., *Therapeutic observation of fire-needle acupuncture for chronic cough after a cold.* Shanghai journal of acupuncture and moxibustion [shang hai zhen jiu za zhi], 2016. **35**(4): p. 411‐412.

93. Qin, J., et al., *Clinical study of strengthen spleen and warm lung on treating chronic cough with throat itching.* Zhong yao cai = Zhongyaocai = Journal of Chinese medicinal materials, 2005. **28**(11): p. 1043-1045.

94. Qin, X., Y. Huo, and L. Mi, *Ginger-partitioned moxibustion for 60 cases of chronic cough caused by exogenous diseases.* Zhongguo zhen jiu = Chinese acupuncture & moxibustion, 2015. **35**(7): p. 674.

95. Wang, H.Y., *46 cases with chronic cough of yin deficiency and dryness-heat pattern treated by modified dryness-clearing and lung-saving decoction.* Henan traditional chinese medicine [henan zhong yi], 2015. **35**(4): p. 823‐824.

**2) No information on cough duration (n = 62)**

1. 甄润平, *自拟鱼腥草黄芩汤治疗儿童上气道咳嗽综合征效果观察.* 社区医学杂志, 2014. **12**(01): p. 45-46.
2. 季坤, et al., *冬病夏治穴位贴敷疗法治疗慢性咳嗽肺脾阳虚证临床疗效观察.* 辽宁中医药大学学报, 2018. **20**(12): p. 76-78.
3. 高晓琴, *经方止嗽散加味治疗300例慢性咳嗽的临床疗效.* 当代医药论丛, 2014. **12**(01): p. 176-177.
4. 高洁群, *用灯台叶颗粒辅助治疗儿童喘息性咳嗽和慢性咳嗽的疗效观察.* 求医问药(下半月), 2013. **11**(05): p. 125-126.
5. 南俊国, *分析小儿肺炎支原体感染后痰湿蕴肺型慢性咳嗽采用加味二陈汤治疗的效果.* 中国医药指南, 2019. **17**(28): p. 165-166.
6. 念小桃, *新升阳益胃汤治疗慢性咳嗽患者的临床研究.* 大家健康(学术版), 2015. **9**(08): p. 31.
7. 董红娟, *麦门冬汤治疗慢性咳嗽临床体会.* 中医药临床杂志, 2016. **28**(09): p. 1248-1249.
8. 梁雪 and 仇志锴, *三拗汤合六君子汤化裁治疗小儿慢性咳嗽的临床疗效.* 临床医药文献电子杂志, 2020. **7**(28): p. 31+41.
9. 李光明, et al., *利肺胶囊治疗慢性咳嗽的临床效果.* 南昌大学学报(医学版), 2014. **54**(07): p. 77-78+81.
10. 李英格 and 其木格, *蒙药复方冬青叶胶囊治疗慢性咳嗽240例疗效观察.* 中国民族医药杂志, 2010. **16**(07): p. 64.
11. 李程新, et al., *中西医结合治疗胃食管返流性咳嗽60例.* 中华保健医学杂志, 2015. **17**(06): p. 495-496.
12. 李萍 and 祁钰, *强力枇杷胶囊与牛黄蛇胆川贝液治疗慢性咳嗽疗效比较.* 青海医药杂志, 2004(09): p. 46-47.
13. 林柳廷, *小儿支原体肺炎后慢性咳嗽应用养阴清肺汤联合孟鲁司特治疗的效果.* 人人健康, 2020(08): p. 103.
14. 李杨, 谢钧, and 温伟波, *宣肺降气汤治疗慢性咳嗽临床观察.* 吉林中医药, 2008(08): p. 573.
15. 梅少群, *兰索拉唑等三联药治疗反流性食管炎引起的慢性咳嗽临床价值研究.* 实用糖尿病杂志, 2016. **12**(05): p. 47-48.
16. 孟晓凤, *艾灸肺俞穴治疗肺结核慢性咳嗽的效果与护理.* 光明中医, 2021. **36**(21): p. 3695-3697.
17. 仕丽, et al., *清宣止咳方外敷治疗慢性咳嗽30例.* 中国中医药现代远程教育, 2012. **10**(17): p. 10-11.
18. 徐爱民, *止嗽散加减合苓甘五味姜辛汤用于慢性咳嗽治疗的疗效研究.* 中西医结合心血管病电子杂志, 2016. **4**(18): p. 158+160.
19. 施红伟, *调脾胃法治疗慢性咳嗽临床观察.* 内蒙古中医药, 2015. **34**(12): p. 20-21.
20. 王建华 and 谭庆华, *参苓白术散汤剂联合阿莫西林克拉维在小儿慢性咳嗽的疗效分析.* 湖北科技学院学报(医学版), 2016. **30**(05): p. 414-416.
21. 王曼曼, *滋阴止咳汤治疗小儿慢性咳嗽临床观察.* 光明中医, 2019. **34**(16): p. 2508-2510.
22. 王文红, *经方止嗽散加味治疗慢性咳嗽62例临床观察.* 世界最新医学信息文摘, 2019. **19**(40): p. 194+197.
23. 王首. *加味黄元御下气汤治疗小儿慢性咳嗽50例临床疗效观察*. in *中华中医药学会儿科分会第三十一次学术大会*. 2014. 中国云南昆明.
24. 王首, et al., *调理中气中药对小儿慢性咳嗽远期疗效随访观察.* 深圳中西医结合杂志, 2016. **26**(14): p. 46-47.
25. 王首, et al. *调理中气治疗小儿慢性咳嗽的中医证象量化疗效评价及意义*. in *第二十次全国儿科中西医结合学术会议*. 2016. 中国陕西西安.
26. 王旭 and 郭楠, *贝蒌止嗽散治疗小儿慢性咳嗽干咳类型疗效观察.* 中国中西医结合儿科学, 2016. **8**(03): p. 319-321.
27. 王莹, *玉屏风散加减治疗慢性咳嗽的临床研究.* 中国现代药物应用, 2016. **10**(13): p. 33-34.
28. 姚燕琴, *中药内服辨治肝火犯肺型小儿咳嗽临床研究.* 浙江中医杂志, 2019. **54**(03): p. 171-172.
29. 于世华, *养阴清肺汤配合孟鲁司特治疗小儿支原体肺炎后慢性咳嗽的疗效.* 中国社区医师, 2018. **34**(10): p. 124-125.
30. 于阅尽, et al., *中医定向透药治疗仪辅助治疗儿童上气道咳嗽综合征的疗效.* 中国医疗设备, 2019. **34**(S1): p. 37-38.
31. 章秀辉, *辛芩颗粒在上气道咳嗽综合征中的临床应用心得.* 内蒙古中医药, 2013. **32**(18): p. 32.
32. 庄岚清, et al., *搜风止咳方治疗职业性咳嗽的临床研究.* 中医临床研究, 2017. **9**(12): p. 53-56.
33. 程桯, *120例慢性咳嗽的中医治疗临床研究.* 中国当代医药, 2009. **16**(14): p. 95-96.
34. 曾慧频, et al., *艾灸肺俞穴治疗肺结核慢性咳嗽的效果与护理.* 中华现代护理杂志, 2012(21): p. 2534-2535.
35. 戚本明, *苏黄止咳胶囊治疗上呼吸道咳嗽综合征的疗效观察.* 云南医药, 2013. **34**(05): p. 393-395.
36. 彭伟, *苓甘五味姜辛汤合止嗽散加减治疗慢性咳嗽临床效果及安全性评价.* 中国社区医师, 2018. **34**(21): p. 96-97.
37. 何强, *滋阴止咳汤治疗小儿慢性咳嗽的临床研究.* 光明中医, 2017. **32**(20): p. 2960-2961.
38. 叶焰 and 里自然, *止嗽散治疗慢性咳嗽60例临床观察.* 中国医药导报, 2009. **6**(29): p. 86+89.
39. 冯发扬, *滋阴止咳汤治疗小儿慢性咳嗽临床效果观察.* 人人健康, 2019(10): p. 111.
40. 刘靖, et al., *疏风宣肺法治疗慢性咳嗽30例的临床观察.* 临床医药文献电子杂志, 2017. **4**(03): p. 533.
41. 刘茜, *养阴祛风方对咳嗽变异型哮喘（CVA）肺阴亏虚证的临床疗效评价研究*. 2020, 南京中医药大学.
42. 单宝霞 and 刘廷胜, *宣肺汤治疗慢性咳嗽40例.* 实用中医内科杂志, 2011. **25**(08): p. 40-41.
43. 张峰, *止嗽散加减治疗慢性咳嗽80例临床观察.* 世界最新医学信息文摘, 2017. **17**(65): p. 192.
44. 张一, *苏黄止咳胶囊对慢性咳嗽的疗效分析.* 世界最新医学信息文摘, 2015. **15**(A0): p. 101.
45. 张表一, *杏仁薏仁鸡蛋汤治好了我的慢性咳嗽.* 求医问药, 2013(10): p. 44-45.
46. 张诗竹, et al., *治疗阴虚肺燥型慢性咳嗽经验处方的疗效评价的研究.* 健康之路, 2017. **16**(05): p. 35.
47. 杨若俊, et al. *止嗽散联合孟鲁司特治疗小儿肺炎支原体感染后慢性咳嗽临床观察*. in *中华中医药学会儿科分会第三十次学术大会*. 2013. 中国山东济南.
48. 杨洁, *中药穴位贴敷治疗慢性咳嗽120例.* 中国中医药科技, 2014. **21**(04): p. 397.
49. 贾温春, *辛汤合止嗽散加减治疗慢性咳嗽临床效果分析.* 世界最新医学信息文摘, 2018. **18**(65): p. 159.
50. 赵东凯 and 王檀, *应用乌梅汤治疗慢性咳嗽(脏气虚寒型)60例临床观察.* 中国医学工程, 2010. **18**(03): p. 147.
51. 赵东凯 and 王檀, *应用清宣止咳汤治疗慢性咳嗽(脾经伏火型)70例临床观察.* 中国医学工程, 2011. **19**(09): p. 147+151.
52. 边玉玲, *肃降肺胃法治疗胃食管反流性咳嗽的临床观察.* 中国卫生标准管理, 2015. **6**(08): p. 56-57.
53. 邹子娟, *玄参升麻汤治疗小儿慢性咳嗽的效果研究.* 中国卫生标准管理, 2015. **6**(22): p. 134-135.
54. 郑蛟东, *止嗽散加减治疗慢性咳嗽临证体会.* 四川中医, 2013. **31**(11): p. 105-106.
55. 陈小伟, et al., *止嗽散加减治疗肺结核慢性咳嗽80例.* 中国中医药科技, 2015. **22**(05): p. 538.
56. 马晶, *止嗽散合三拗汤加减治疗慢性咳嗽的效果及安全性评价.* 中西医结合心血管病电子杂志, 2019. **7**(24): p. 162+173.
57. 黄戍成, 童红卫, and 叶圣雅, *射干麻黄汤治疗肺癌根治术后慢性咳嗽的临床观察.* 浙江中医药大学学报, 2012. **36**(04): p. 398+404.
58. 黄载峰, *滋阴止咳汤治疗小儿慢性咳嗽的效果评价.* 临床医药文献电子杂志, 2019. **6**(19): p. 150.
59. ChiCtr, *Standardized research for acupoint application of traditional Chinese medicine in the treatment of children with chronic cough.* https://trialsearch.who.int/Trial2.aspx?TrialID=ChiCTR1800019299, 2018.
60. Mukaida, K., et al., *A pilot study of the multiherb Kampo medicine bakumondoto for cough in patients with chronic obstructive pulmonary disease.* Phytomedicine, 2011. **18**(8‐9): p. 625‐629
61. Wang, L., et al., *Efficacy of acupuncture combined with Chinese herb in refractory chronic cough.* American journal of respiratory and critical care medicine, 2014. **189**(no pagination).
62. Watanabe, N., C. Gang, and T. Fukuda, *The effects of bakumondo-to (mai-men-dong-tang) on asthmatic and non-asthmatic patients with increased cough sensitivity.* Nihon Kokyūki Gakkai zasshi = the journal of the Japanese Respiratory Society, 2004. **42**(1): p. 49-55.

**3) Accompanied by other diseases not related to cough (n = 11)**

1. 姜利强 and 张雪娟, *参松养心胶囊对室性期前收缩相关慢性咳嗽的临床疗效研究.* 临床医药文献电子杂志, 2020. **7**(10): p. 150-151.
2. 姜永红, et al., *中药联合耳穴贴压治疗以慢性咳嗽为主症的儿童多发性抽动症.* 世界中西医结合杂志, 2018. **13**(11): p. 1556-1559.
3. 唐洁芬 and 余培煌, *养阴清肺汤联合孟鲁司特治疗小儿支原体感染肺炎后慢性咳嗽临床观察.* 四川中医, 2016. **34**(08): p. 72-75.
4. 王友梅 and 张小丽, *口服加巴喷丁胶囊治疗慢性咳嗽3例疗效分析.* 浙江医学, 2017. **39**(07): p. 572-574.
5. 楚玉波, 宋桂华, and 孙萌萌, *加味升降散治疗小儿慢性咳嗽并抽动症验案举隅.* 中国中西医结合儿科学, 2014. **6**(06): p. 502-503.
6. 张元兵, 徐超, and 刘良徛, *半夏厚朴汤治疗阻塞型睡眠呼吸暂停低通气综合征相关性慢性咳嗽初探.* 中华中医药杂志, 2017. **32**(10): p. 4522-4524.
7. 杨慧, *自拟麻蝉止咳汤治疗风盛挛急夹热型CVA的临床疗效及对血清IL-5的影响*. 2018, 黑龙江中医药大学.
8. 黄金凤, *苓甘五味姜辛汤联合止咳散治疗慢性咳嗽伴睡眠障碍的疗效及对患者症状改善及睡眠质量的影响.* 世界睡眠医学杂志, 2022. **9**(03): p. 426-428.
9. Chen, J., et al., *TCM treatment of interstitial pneumonia with chronic cough - A case report.* Journal of Traditional Chinese Medicine, 2003. **23**(3): p. 170-171.
10. Hong, G., et al., *Effects of mixture of IVY leaves and coptidis rhizome in patients with chronic bronchitis and/or bronchiectasis.* Respirology (Carlton, Vic.), 2019. **24**: p. 103‐.
11. Yu, L.H., *Clinical analysis on Sanzi Yangqin Decoction and Erchen Decoction in the treatment of acute exacerbations of chronic bronchitis for 60 cases.* Guangming journal of chinese medicine [guang ming zhong yi], 2015. **30**(9): p. 1888‐1890.

**4) Not about only EATM (n = 8)**

1. 李君君, *中药离子导入佐治小儿慢性咳嗽鼻后滴漏综合征的临床疗效观察*. 2007, 福建中医学院.
2. 樊锦青, et al., *温针灸结合穴位注射弥可保治疗慢性咳嗽的临床观察.* 南京医科大学学报(自然科学版), 2014. **34**(09): p. 1253-1254.
3. 石磊, *慢性咳嗽67例病因及临床疗效分析.* 现代诊断与治疗, 2013. **24**(01): p. 206-207.
4. 宋占杰, *止嗽散合转移因子治疗慢性咳嗽临床观察.* 光明中医, 2013. **28**(08): p. 1655-1656.
5. 丁申 and 边逊, *慢性咳嗽的中药外用体会.* 中国中西医结合儿科学, 2010. **2**(01): p. 80-81.
6. 刘殿玉 and 王秀坤, *中西药结合治疗儿童上气道咳嗽综合征临床疗效分析.* 辽宁中医药大学学报, 2012. **14**(11): p. 195-196.
7. 陈红, et al., *清肺止咳方加炎琥宁治疗慢性咳嗽疗效观察.* 湖北中医药大学学报, 2012. **14**(03): p. 47-48.
8. Wang, L., M.Y. Liang, and T.T. Lu, *Nursing of integrated medicine for children with chronic cough.* Chinese medicine modern distance education of china [zhong guo zhong yi yao xian dai yuan cheng jiao yu], 2015. **13**(6): p. 113‐114.

**5) Review articles (n = 26)**

1. 高锋 and 高玉娟, *李孔定治疗慢性咳嗽的经验.* 四川中医, 2010. **28**(07): p. 6-7.
2. 郭燕, *盛丽先教授运用养阴清肺汤治疗小儿肺炎支原体感染后慢性咳嗽经验.* 中医儿科杂志, 2010. **6**(02): p. 10-11.
3. 李军, *透法治疗慢性咳嗽验案4则.* 江苏中医药, 2017. **49**(08): p. 41-43.
4. 傅峪松 and 何志坚, *止嗽散加减治验慢性咳嗽2则.* 北京中医药, 2008(11): p. 878.
5. 徐波 and 苗青, *苗青运用小柴胡汤合止嗽散治疗慢性咳嗽验案2则.* 湖南中医杂志, 2016. **32**(08): p. 118-119.
6. 余松 and 张立山, *小柴胡汤治疗慢性咳嗽验案2则.* 光明中医, 2017. **32**(20): p. 3014-3015.
7. 王春辉, *祛风法在儿童慢性咳嗽中的应用.* 江苏中医药, 2020. **52**(05): p. 72-74.
8. 王熙芝, et al., *盛丽先运用六味汤治疗小儿慢性咳嗽三则.* 浙江中西医结合杂志, 2017. **27**(05): p. 357-358.
9. 周欣欣, *麦门冬汤加减在呼吸系统疾病中的临床应用与药理价值.* 临床医药文献电子杂志, 2017. **4**(18): p. 3553+3556.
10. 楚玉波, *升降散加味治疗小儿慢性咳嗽临症体会.* 中医临床研究, 2015. **7**(05): p. 103-104.
11. 解潇清, 袁世伟, and 郝瑞芳, *中医药治疗儿童慢性咳嗽验案2则.* 湖南中医杂志, 2018. **34**(04): p. 107-108.
12. 刘卿 and 胡淑萍, *杏苏散加减治疗小儿慢性咳嗽验案二则.* 浙江中医杂志, 2019. **54**(01): p. 56.
13. 刘威 and 欧江琴, *戴永生辨体论治慢性咳嗽临证经验.* 中国民间疗法, 2020. **28**(20): p. 27-29.
14. 卢传坚 and 邓静文, *慢性咳嗽,勿忘止嗽散.* 家庭药师, 2011(01): p. 36-41.
15. 吕萌 and 刘建博, *刘建博运用温胆汤治疗慢性咳嗽验案4则.* 江苏中医药, 2014. **46**(01): p. 53-54.
16. 孙国明, 王艳云, and 李晓春, *四逆散加减治疗慢性咳嗽验案3则.* 河北中医, 2012. **34**(08): p. 1165-1166.
17. 张照乾, *慢性咳嗽的中医药治疗研究*. 2008, 南京中医药大学.
18. 杨丽 and 石磊, *止嗽散加味治疗慢性咳嗽的临床体会.* 内蒙古中医药, 2011. **30**(08): p. 60.
19. 纪才书, *金沸草散治疗慢性咳嗽经验.* 上海中医药杂志, 2013. **47**(05): p. 75.
20. 陈蓓华, *辛开苦降法论治小儿慢性咳嗽.* 中华中医药学刊, 2013. **31**(09): p. 2036-2037.
21. 陈拥军, et al., *金沸草散治疗慢性咳嗽.* 实用中医内科杂志, 2014. **28**(02): p. 134-136.
22. 马冲, et al., *王书臣从肺脾肾论治慢性咳嗽验案3则.* 江苏中医药, 2019. **51**(04): p. 46-48.
23. 黄炜萍 and 范发才, *从肺胃论治慢性咳嗽高敏综合征验案1则.* 湖南中医杂志, 2021. **37**(07): p. 79-80.
24. 齐永福, 王胜, and 毛照海, *毛照海主任医师治疗小儿慢性咳嗽经验.* 中国中西医结合儿科学, 2011. **3**(05): p. 408-409.
25. Wen, B.L., et al., *[Analysis on acupoint prescription for acupoint sticking therapy of treating winter diseases in summer for preventing and curing chronic cough and asthma].* Zhongguo zhen jiu = Chinese acupuncture & moxibustion, 2010. **30**(8): p. 647-652.
26. Zou, J., L. Pang, and X. Lv, *Network Pharmacology Systematically Uncovers Multiple Mechanisms of Zhisousan () for Treatment of Chronic Cough.* European Journal of Integrative Medicine, 2020. **35**.

**6) Duplicate (n = 2)**

1. 姜成, *清肺止咳方化裁治疗慢性咳嗽的临床疗效观察*. 2012, 湖北中医药大学.
2. Cui, X., S.M. Wang, and L.Q. Wu, *Sixty-eight cases of child chronic cough treated by moxibustion.* Journal of Traditional Chinese Medicine, 2009. **29**(1): p. 9-10.

**7) Only abstract available without data (n = 4)**

1. 高伟, et al., *自拟疏木运土止咳方结合西医常规疗法治疗胃食管反流性咳嗽临床研究.* 国际中医中药杂志, 2017. **39**(05): p. 420-423.
2. 肖丽春. *止咳利咽汤治疗慢喉痹之慢性咳嗽150例临床观察*. in *中华医学会呼吸病学年会——2013（第十四次全国呼吸病学学术会议）*. 2013. 中国辽宁大连.
3. 蒲昭和, *鲫鱼杏仁汤治慢性喘咳.* 晚霞, 2016(05): p. 56.
4. 何敏仪 and 林俏丽, *四子散药熨法联合超激光治疗仪治疗慢性咳嗽疗效观察.* 按摩与康复医学, 2017. **8**(13): p. 57-58.
