# Supplementary material for: Research status of east Asian traditional medicine treatment for chronic cough: A scoping review
Source: PLoS One. 2024 Feb 8;19(2):e0296898. doi: 10.1371/journal.pone.0296898 (PMC10852285; doi:10.1371/journal.pone.0296898)
Supplement: S3 Appendix — (DOCX) [file pone.0296898.s003.docx]

**S3 Appendix. References of the included studies**

1. 陈奕庆, 李珑. “降气化痰止咳汤”治疗慢性咳嗽266例临床效果观察. 安徽中医学院学报. 1985(01):10-1.

2. Cheng CL. Clinical and experimental research on chronic bronchitis treated with ke chuan ping decoction. Zhong xi yi jie he za zhi = chinese journal of modern developments in traditional medicine. 1991;11(4):203‐5, 195.

3. 夏正飞. 宁嗽汤治疗慢性咳嗽疗效观察. 中国中医药信息杂志. 1997(11):28.

4. 姜志业. 中西医结合治疗咳嗽变异型哮喘56例. 湖北中医杂志. 2003(10):23.

5. Wang J, Chen S, Zhu X. Clinical observation on cough asthma plastor treating patients with chronic bronchitis (type of cold - phlegm) by acupoint application. Journal of emergency in traditional chinese medicine. 2003;12(2):119.

6. 甄穗清, editor 玉屏风散加味治疗小儿鼻后滴漏综合征. 刘弼臣教授从医六十五周年学术思想研讨会暨中华中医药学会儿科分会与全国中医药高等教育学会儿科分会全国学术交流大会; 2004; 中国北京.

7. 高静. 儿童慢性咳嗽病因及治法临床研究 [硕士]: 福建中医学院; 2005.

8. 范伏元, 罗姣利. 自拟疏风宣肺汤治疗慢性咳嗽300例临床观察. 中医药导报. 2006(02):33-4+43.

9. 喻清和, 邱志楠. 中西医结合治疗小儿鼻后滴漏致慢性咳嗽疗效观察. 中医药学刊. 2006(12):2353-4.

10. 段雨. 喉疳散喷敷治疗痰浊血瘀型慢性咳嗽165例. 陕西中医. 2007(10):1334.

11. 卞国本. 苍夷射干汤治疗儿童鼻后滴流综合征60例. 陕西中医. 2007(11):1459-60.

12. 王有广, 宋吉义. 速效定嗽中药配方颗粒治疗慢性咳嗽108例. 新中医. 2007(12):66-7.

13. 殷卫东, 谢友华, 王永年. 双藤汤治疗慢性咳嗽52例. 四川中医. 2007(02):63.

14. 崔霞, 王素梅, 吴力群. 雷火灸治疗小儿慢性咳嗽68例. 四川中医. 2007(11):119-20.

15. 吴铭. 益气固表、健脾润肺法治疗小儿外感后久咳 30 例临床观察. 山西中医学院学报. 2007;8(3):31.

16. 张鸿雁. 六君子汤治疗老年慢性咳嗽41例. 实用中医内科杂志. 2007(03):63.

17. 唐海波, 刘百祥. 穴位敷贴咳嗽膏治疗小儿慢性咳嗽67例. 中国民族民间医药. 2008(06):33+65.

18. 朱永耀. 益气养阴方为主治疗小儿慢性咳嗽53例. 陕西中医. 2008(11):1475-6.

19. 刘进虎, editor 宣通鼻咽汤治疗小儿鼻后滴流综合征30例疗效观察. 甘肃省中医药学会2008年学术年会; 2008; 中国甘肃酒泉.

20. 吕华. 清咽汤治疗慢性咳嗽60例. 陕西中医. 2008(04):398.

21. 毕美芬, 石伟松. 温胆汤治小儿胃食道反流性咳嗽30例. 江西中医药. 2008(09):34-5.

22. 赵立新. 参苓白术散加减治疗慢性咳嗽50例. 实用中医药杂志. 2008(05):286.

23. 赵永华. 肺脾同治法治疗小儿慢性咳嗽（痰热壅肺型）的临床研究 [硕士]: 成都中医药大学; 2008.

24. 陈健. 杏苏散加减治疗鼻后滴漏致慢性咳嗽疗效观察. 内蒙古中医药. 2008(15):11.

25. 韩峰, 张竞之, 刘美英. 慢咳饮治疗慢性咳嗽35例. 中医研究. 2008(08):22-4.

26. 黄纯美. 加味三叶汤治疗鼻后滴流综合征慢性咳嗽临床疗效观察 [硕士]: 广州中医药大学; 2008.

27. 李东, 刘承. 肃降肺胃法治疗胃食管反流性咳嗽的临床观察. 中华中医药杂志. 2009;24(01):90-2.

28. 李来祥, editor 自拟理肺涤痰汤治疗慢性咳嗽体会. 甘肃省中医药学会2009年学术研讨会; 2009; 中国甘肃临洮.

29. 常宗焕. 自拟宁嗽汤治疗小儿慢性咳嗽160例. 陕西中医学院学报. 2009;32(04):47.

30. 王桂华. 三拗汤合止嗽散加减治疗慢性咳嗽30例观察. 实用中医药杂志. 2009;25(04):217.

31. 周晓红. 止嗽散加减治疗慢性咳嗽临床体会. 中国临床医生. 2009;37(02):53-4.

32. 刘继民. 加减泻黄散治疗慢性咳嗽（津伤肺燥）临床研究 [硕士]: 长春中医药大学; 2009.

33. 张蕊. 桑栀黛蛤汤治疗肝火犯肺型慢性咳嗽的临床观察. 中国自然医学杂志. 2009;11(04):268-70.

34. 麦志广, 夏鑫华. 天龙咳喘灵组方加味治疗慢性咳嗽146例. 光明中医. 2009;24(01):56-7.

35. 黄纯美, 刘小虹, 许仕杰. 加味三叶汤治疗鼻后滴流综合征慢性咳嗽30例临床观察. 新中医. 2009;41(04):51-3.

36. 李秀英. 止嗽散加减治疗慢性咳嗽临床体会. 中国民康医学. 2010;22(10):1261.

37. 李秀英. 止嗽散加减治疗慢性咳嗽疗效观察. 中国实用医药. 2010;5(08):151-2.

38. 李晓燕, 石颖. 自拟方治疗阴虚肺燥型慢性咳嗽64例. 中医研究. 2010;23(06):38-9.

39. 史苗颜, 毕小利, 张炜. 平金方治疗慢性咳嗽的临床研究. 上海中医药杂志. 2010;44(11):54-6.

40. 徐宁. 苍屏散加减治疗小儿UACS所致慢性咳嗽的临床疗效观察 [硕士]: 成都中医药大学; 2010.

41. 於志娟. 养阴清肺汤加减治疗小儿上气道咳嗽综合征75例疗效观察. 内蒙古中医药. 2010;29(22):10.

42. 王彩霞. 防哮冲剂治疗小儿慢性咳嗽（气阴两虚型）的临床研究 [硕士]: 黑龙江中医药大学; 2010.

43. 王洪白. 止嗽散加减治疗慢性咳嗽46例观察. 实用中医药杂志. 2010;26(07):468-9.

44. 郁晓维, 王明明. 加味玉屏风散治疗小儿慢性咳嗽肺脾两虚证疗效评价. 中医儿科杂志. 2010;6(01):23-7.

45. 秦莹, 张思文, 顾炳岐. 中药内外同治法治疗鼻后滴流综合征致慢性咳嗽的临床研究. 上海中医药杂志. 2010;44(05):57-9.

46. 支献峰. 益气养阴法治疗慢性咳嗽52例疗效观察. 贵阳中医学院学报. 2010;32(06):43-5.

47. 吴肖妮, 吴庆福. 中医治疗鼻后滴漏综合征引起儿童慢性咳嗽40例. 中医儿科杂志. 2010;6(01):29-30.

48. 孙恒宇. 脾经伏火论治疗慢性咳嗽（脾经伏火）的临床研究 [硕士]: 长春中医药大学; 2010.

49. 张淑英. 小陷胸汤合止嗽散治疗慢性咳嗽临床观察. 辽宁中医杂志. 2010;37(08):1521-2.

50. 杨春元. 清降疏肺汤治疗慢性咳嗽100例观察. 实用中医药杂志. 2010;26(05):303.

51. 谭捷, 吕献青. 应用三仁汤从湿论治慢性咳嗽临床观察. 四川中医. 2010;28(11):83-4.

52. 郑锐平. 加味香砂六君子颗粒治疗小儿肺脾气虚型慢性咳嗽的临床研究 [硕士]: 黑龙江中医药大学; 2010.

53. 顾莹莹, 张晓利, editors. 中药配方颗粒和西药联用治疗小儿慢性咳嗽600例疗效观察. 吉林省第六届科学技术学术年会; 2010; 中国吉林长春.

54. 丘梅清. 加味旋复代赭汤治疗慢性咳嗽30例. 广州医药. 2011;42(05):48-9.

55. 李赞峰, 刘玉萍, 高明. 金玄利咽颗粒治疗成人慢性咳嗽的疗效观察. 临床合理用药杂志. 2011;4(34):74.

56. 范平, 陈革妃. 温胆汤治疗慢性咳嗽胃气上逆证30例. 河南中医. 2011;31(03):267.

57. 凡怀亲. 从寒从虚治疗慢性咳嗽35例. 中医药临床杂志. 2011;23(09):776-7.

58. 余燕. 苓甘五味姜辛汤合止嗽散加减治疗慢性咳嗽临床观察. 中国当代医药. 2011;18(12):110-1.

59. 王皓. 参苓白术散治疗鼻后滴漏综合征的疗效观察. 四川中医. 2011;29(05):105-6.

60. 左明晏, 李高兵, 范世友. 中西医结合治疗慢性咳嗽30例. 光明中医. 2011;26(12):2514-5.

61. 胡崇静. 加味止嗽颗粒治疗风邪恋肺型慢性咳嗽的临床疗效观察 [硕士]: 广州中医药大学; 2011.

62. 冯江江, 高洁. 止嗽散加减治疗老年慢性咳嗽40例. 山西中医. 2011;27(11):17+9.

63. 吴眉, 张发明, 王艳. 止嗽化痰汤治疗慢性咳嗽450例疗效观察. 医学信息(中旬刊). 2011;24(09):4967.

64. 杨志强. 麦门冬汤加减治疗慢性咳嗽40例. 实用中医内科杂志. 2011;25(12):23-5.

65. 杨辉, 武志娟, 张大鹏. 中药二仙饮治疗儿童慢性咳嗽临床观察. 医学研究杂志. 2011;40(05):60-2.

66. 谌莹. 加味止嗽散治疗小儿感染后慢性咳嗽的临床观察. 中医药导报. 2011;17(04):44-6.

67. 赵丽芸, 陈宁. 清燥救肺汤加减方治疗鼻后滴流综合征疗效观察. 中国民族民间医药. 2011;20(07):73-4.

68. 郑芳. 慢咳散治疗老年慢性咳嗽125例临床观察. 浙江中医杂志. 2011;46(06):421.

69. 顾明达, editor 中药配方颗粒治疗儿童风热型UACS的临床观察. 第28次全国中医儿科学术大会暨2011年名老中医治疗（儿科）疑难病临床经验高级专修班; 2011; 中国浙江宁波.

70. 管志伟, 代运磊, 于素平, 刘玉慧, 宋桂华. 活血通络中药佐治儿童慢性咳嗽30例疗效观察. 中国中西医结合儿科学. 2012;4(03):208-9.

71. 管奕婷. 止嗽散加减治疗慢性咳嗽80例临床观察. 实用中医内科杂志. 2012;26(15):8+10.

72. 李隆庆. 桔前汤治疗小儿慢性咳嗽30例疗效观察. 河北中医. 2012;34(05):680.

73. 李永昌. 自拟旋覆代赭连叶汤治疗胃食管反流性咳嗽体会. 中国中医药信息杂志. 2012;19(08):83.

74. 潘奇林, 余洋, 李春玲. 加味六君子汤治疗慢性咳嗽40例疗效观察. 云南中医中药杂志. 2012;33(05):82.

75. 潘文超, 赵蓓, 史锁芳. 上气道咳嗽综合征风痰恋肺证中医药临床治疗观察. 中医临床研究. 2012;4(08):11-2.

76. 付泽伟. 中药治疗慢性咳嗽疗效观察. 临床合理用药杂志. 2012;5(04):16-7.

77. 徐菁. 沙参麦冬汤合芍药甘草汤治疗肺炎支原体感染后小儿慢性咳嗽60例. 中国中医药科技. 2012;19(02):101.

78. 石效平, 王巍, editors. 中药浓煎剂小儿止咳方治疗儿童慢性咳嗽的临床应用体会. 第二十九次全国中医儿科学术大会暨“小儿感染性疾病的中医药防治”培训班; 2012; 中国湖南张家界.

79. 徐洁. 针灸治疗鼻后滴流综合征引起慢性咳嗽60例. 浙江中医药大学学报. 2012;36(01):80-1.

80. 朱越. 沙参麦冬汤加减辨治慢性咳嗽68例. 实用中医内科杂志. 2012;26(18):21-2.

81. 肖钦, 居来提·艾买提. 止嗽散合三拗汤加减治疗慢性咳嗽的临床疗效. 实用中医内科杂志. 2012;26(07):24-5.

82. 农志飞, 蒙美禄. 小柴胡汤合苓桂术甘汤治疗儿童慢性咳嗽临床观察. 辽宁中医杂志. 2012;39(10):1999-2000.

83. 刘超. 玉屏风散合四磨汤加减治疗慢性咳嗽60例. 中外医疗. 2012;31(25):77-8.

84. 刘慧兰, 张洛萍. 加味止嗽散治疗小儿慢性咳嗽疗效分析. 中国中医药信息杂志. 2012;19(12):69-70.

85. 刘洁华, 郭永坚. 自拟疏风通窍利咽汤治疗上气道咳嗽综合征36例. 中国民间疗法. 2012;20(01):34-5.

86. 吴梦晖, 张竹君. 通窍鼻炎颗粒辅助治疗儿童上气道咳嗽综合征的近期疗效观察. 中国医药指南. 2012;10(24):50-1.

87. 张喆. 肺胃相关性慢性咳嗽的临床特征分析及治疗研究 [博士]: 北京中医药大学; 2012.

88. 杨加禄, 杨晓. 姜辛化痰止咳方治疗慢性咳嗽51例疗效分析. 实用中医内科杂志. 2012;26(06):32-3.

89. 杨宇华, 李俊滔. 针刺结合皮内针治疗妊娠期慢性咳嗽1例. 新中医. 2012;44(06):145-6.

90. 简小云, 黄兆祈, 赖昕, 陈伟云. 小柴胡汤合升降散治疗慢性咳嗽32例. 实用中医内科杂志. 2012;26(12):23-4+6.

91. 谢嘉嘉, 杨从意, 黄寅銮, 曾小玲, 林国彬. 沙参麦冬汤加减治疗慢性咳嗽患者的临床观察. 中国医药指南. 2012;10(06):43-4.

92. 邓剑英. 苗药联合左金丸治疗胃食管反流性咳嗽随机对照临床观察. 实用中医内科杂志. 2012;26(17):11-2.

93. 陈冬梅. 苓桂术甘汤加味治疗慢性咳嗽临床研究. 中医学报. 2012;27(12):1641-2.

94. 陈小芳, 胡晓岚. 中药方剂治疗小儿慢性咳嗽50例临床观察. 中国社区医师(医学专业). 2012;14(32):195.

95. 颜蔓仪. 止嗽散合三拗汤加减治疗慢性咳嗽疗效观察. 亚太传统医药. 2012;8(02):73-4.

96. 黄芳, 周芳. 玉屏风颗粒联合孟鲁司特钠治疗儿童反复呼吸道感染后咳嗽的疗效观察. 海峡药学. 2012;24(10):177-80.

97. 曲婧. 新升阳益胃汤治疗慢性咳嗽（中气不足，肺气不清证）临床研究 [硕士]: 长春中医药大学; 2013.

98. 郭文英, 任体祥. 沙参麦门冬汤加减治疗儿童慢性咳嗽300例临床观察. 云南中医中药杂志. 2013;34(03):36.

99. 管奕婷. 肃降肺胃法治疗胃食管反流性咳嗽的临床观察 [硕士]: 广州中医药大学; 2013.

100. 梁山. 中药辛鹅鼻喷剂联合氯苯那敏治疗上气道咳嗽综合征及对慢性鼻-鼻窦炎治疗作用探讨. 辽宁中医杂志. 2013;40(10):2043-4.

101. 李晓燕, 吕红玲, 童晓云. 养阴润肺方治疗慢性咳嗽的临床研究. 中医学报. 2013;28(05):648-9.

102. 万丽玲, 杨铁柱. 疏风降气止咳汤对慢性咳嗽气道高反应性影响的临床研究. 时珍国医国药. 2013;24(07):1669-70.

103. 梅明, 郭丽. 蚕蝉颗粒治疗儿童上气道咳嗽综合征45例临床观察. 河北中医. 2013;35(11):1640-1.

104. 文孟先. 肺力咳胶囊治疗慢性咳嗽的证效关系研究 [硕士]: 山东中医药大学; 2013.

105. 符艳. 疏风宣肺汤治疗嗜酸粒细胞性支气管炎风盛挛急证的临床疗效观察 [硕士]: 湖南中医药大学; 2013.

106. 徐亚琴. 加味止嗽散治疗小儿慢性咳嗽临床观察. 大家健康(学术版). 2013;7(11):75.

107. 宋芊. 慢性咳嗽证候特征与“温润辛金培本”法应用研究 [博士]: 北京中医药大学; 2013.

108. 宋超一, 王秀英. 滋肾养肺止咳汤治疗慢性咳嗽38例. 中国中医药现代远程教育. 2013;11(13):26-7.

109. 阮加飞. 麦门冬汤合止嗽散治疗慢性咳嗽36例. 江苏中医药. 2013;45(01):35.

110. 殷文银, 尹玉平. 柔肝理气止咳方从肝论治慢性支气管炎慢性咳嗽慢性迁延期对照观察. 实用中医内科杂志. 2013;27(04):8-9.

111. 周文瑾, 覃冠锻, 彭清华, 莫炼, 梁志成, 侯涛. 小青龙汤加减治疗鼻后滴漏综合征的疗效观察. 四川中医. 2013;31(04):122-3.

112. 朱瑞华, 袁桂洪, 王跃, 王文娟, 杨云, 张书华. 旋覆泻心止咳汤治疗胃食管反流性咳嗽的临床研究. 辽宁中医杂志. 2013;40(08):1638-9.

113. 周红琼. 夷芎麻杏汤治疗小儿上气道咳嗽综合征（风痰恋肺证）的临床疗效观察 [硕士]: 成都中医药大学; 2013.

114. 崔华. 中药治疗小儿肝火犯肺型慢性咳嗽38例. 河南中医. 2013;33(11):1954-5.

115. 何欢. 中医治疗小儿过敏相关性慢性咳嗽初步研究 [硕士]: 成都中医药大学; 2013.

116. 卢敬东. 小青龙汤加减治疗小儿外邪里饮证慢性咳嗽41例. 河北中医. 2013;35(02):227-8.

117. 卢保强, 范良, 潘小丹. 半夏泻心汤加减治疗慢性咳嗽临床研究. 中医学报. 2013;28(08):1118-9.

118. 杨芝贵. 清肺止咳汤治疗儿童感染后慢性咳嗽53例临床观察. 河北中医. 2013;35(07):985-6.

119. 杨胜利, 颜昭君, 赵家亮. 活血利咽方治疗慢性咳嗽疗效观察. 中国中医药信息杂志. 2013;20(12):72-3.

120. 罗志泉. 自拟青黛三百汤治疗慢性咳嗽84例临床分析. 四川中医. 2013;31(09):80-1.

121. 茆俊卿. 中药颗粒剂(止咳1号)治疗慢性咳嗽的临床研究. 中医临床研究. 2013;5(17):6-7.

122. 谢木军, 谢作权. 桂枝加厚朴杏子汤治疗慢性咳嗽278例. 实用中医药杂志. 2013;29(01):14.

123. 赵淑平. 止嗽散加减配合穴位贴敷治疗慢性咳嗽50例. 中国医药指南. 2013;11(23):281.

124. 郑世铎, 刘海涛. 半夏厚朴汤合玄麦甘桔汤治疗慢性咳嗽100例临床观察. 甘肃医药. 2013;32(05):386-7.

125. 郑岳花. 桑百杏龙汤治疗慢性咳嗽53例疗效观察. 新中医. 2013;45(02):57-8.

126. 陈淑婉. 中西医结合治疗慢性咳嗽60例. 中国中医药现代远程教育. 2013;11(15):55.

127. 陈照南, 陈昭玲, 宋天云, 惠萍. 百合固金汤加味治疗肺阴虚型慢性咳嗽40例临床观察. 湖南中医杂志. 2013;29(06):14-6.

128. 顾惠英. 止嗽散治疗慢性咳嗽随机平行对照研究. 实用中医内科杂志. 2013;27(05):39-40.

129. 龙江. 苏黄止咳胶囊治疗慢性咳嗽43例. 中国中医药现代远程教育. 2013;11(20):32-3.

130. Jin PC, Fang XL. Wentong needling method in the treatment of 30 cases with long-term cough after common cold. World Journal of Acupuncture - Moxibustion. 2013;23(3):62-5.

131. 陶应新. 止嗽散治疗慢性咳嗽50例临床观察. 实用中医内科杂志. 2014;28(01):24-5.

132. 李冬梅, 周维维, 曹骅. 祛风止咳汤结合西医治疗小儿慢性咳嗽30例疗效观察. 中国优生优育. 2014;20(01):25-7.

133. 李育祥. 苓甘五味姜辛汤合止嗽散加减治疗慢性咳嗽的思路探讨. 中国社区医师. 2014;30(05):86-7.

134. 林宏, 张智琳. 小青龙汤加减治疗慢性咳嗽的临床体会. 现代医院. 2014;14(08):69-70.

135. 荀春铮, 王孟清. 小儿久咳方治疗儿童感染后慢性咳嗽110例临床观察. 中医药导报. 2014;20(03):101-2.

136. 王琦芬. 自拟温阳汤治疗阳虚质型小儿慢性咳嗽疗效观察. 中国处方药. 2014;12(07):130.

137. 王世强. 橘红痰咳颗粒联合西药治疗慢性咳嗽临床观察. 新中医. 2014;46(09):50-1.

138. 汪凤仙, 汪永和. 加味四逆散治疗胃食管反流病所致慢性咳嗽临床观察. 新中医. 2014;46(07):59-60.

139. 姚俊丽, 高志强. 止嗽散加味(鼻肺同治)治疗小儿慢性咳嗽的临床体会. 湖北中医杂志. 2014;36(02):51-2.

140. 魏文君. 强力枇杷胶囊联合头孢呋辛酯片治疗慢性咳嗽110例临床观察. 北京中医药. 2014;33(10):764-5.

141. 朱沈芳. 延年半夏汤治疗慢性咳嗽25例疗效观察. 浙江中医杂志. 2014;49(10):724.

142. 支艳, 张文龙, 马新英, 董静, 马建伟, 魏汉林, et al. 滋阴清热法为主治疗慢性咳嗽临床研究. 中医临床研究. 2014;6(31):12-3.

143. 崔娣. 疏风宣肺汤联合西药治疗风邪恋肺型慢性咳嗽随机平行对照研究. 实用中医内科杂志. 2014;28(09):90-2.

144. 郝尧坤, 刘佳, 周福生. 周福生教授补益脾肺法治疗慢性胃食管反流性咳嗽. 光明中医. 2014;29(01):39-40.

145. 何胜尧, 李秀兰, 吴仙娜, 曾明华. 四子散外敷佐治儿童慢性咳嗽临床观察. 现代中西医结合杂志. 2014;23(17):1880-1+924.

146. 何远方, 陶蕾, 施江艳. 中药热奄包治疗慢性咳嗽56例观察. 中医临床研究. 2014;6(30):29-30.

147. 叶振东. 中医治疗慢性咳嗽临床疗效观察. 中医临床研究. 2014;6(14):122-3.

148. 刘媛媛, 艾宙, 张倩如, 王雪玲. 穴位贴敷结合点刺四缝穴治疗小儿慢性咳嗽临床观察. 上海针灸杂志. 2014;33(10):920-1.

149. 刘怡静. 红外止咳贴穴位贴敷为主的中医康复方案在社区慢性咳嗽患者中的应用. 临床合理用药杂志. 2014;7(06):133-4.

150. 刘东宇. 柴朴止咳汤治疗慢性咳嗽（肝郁气滞证）临床观察 [硕士]: 长春中医药大学; 2014.

151. 刘学春, 王流云. 用止咳散加减治疗小儿慢性咳嗽的效果观察. 当代医药论丛. 2014;12(13):143.

152. 刘苏伟. 玄参升麻汤治疗小儿慢性咳嗽的临床疗效研究 [硕士]: 北京中医药大学; 2014.

153. 刘辉, 孙子凯, 朱佳. 益气疏风法治疗慢性咳嗽的临床疗效及对小气道功能减退的影响. 吉林中医药. 2014;34(08):812-5.

154. 卢保强, 潘小丹. 半夏泻心汤联合三联药物疗法治疗顽固性慢性咳嗽的效果及机制. 中国中医基础医学杂志. 2014;20(08):1087-8+100.

155. 孙建, 侯海慧, 曹拥军. 杏苏颗粒治疗慢性咳嗽40例临床观察. 黑龙江中医药. 2014;43(06):22-3.

156. 张娟, 朱云丽, 王宝玉. 四逆化痰汤治疗慢性咳嗽39例. 陕西中医. 2014;35(11):1488-9.

157. 张军城, 李耀辉, 刘莉君. 穴位贴敷法治疗肺阳虚型慢性咳嗽46例. 陕西中医. 2014;35(07):895.

158. 怀保健, 王莉, 张学军. 泻肺止咳汤加减治疗咳嗽变异型哮喘50例临床观察. 中医临床研究. 2014;6(28):67-8.

159. 杨玲. 桑杏汤+养阴清肺汤在新加坡地区治疗慢性咳嗽（燥邪犯肺证）的临床疗效观察 [硕士]: 南京中医药大学; 2014.

160. 杨春玲, 周颖, 李显春. 李显春主任医师运用止咳二号方治疗小儿慢性咳嗽经验. 中国中西医结合儿科学. 2014;6(04):308-9.

161. 苏成程, 唐艳芬, 章匀. 降气止咳免煎颗粒治疗风盛挛急型慢性咳嗽30例. 现代中医药. 2014;34(05):17-8.

162. 许多敏. 加味二陈汤治疗小儿痰湿蕴肺型慢性咳嗽的临床研究 [硕士]: 广州中医药大学; 2014.

163. 郑文龙. 二陈汤+四君子汤加味治疗46例小儿慢性咳嗽疗效观察. 中医临床研究. 2014;6(10):98-9.

164. 钟丹. 中药敏咳煎对阴虚肺燥型CVA患者气道神经源性炎症影响及生活质量干预研究 [博士]: 成都中医药大学; 2014.

165. 陆彪. 益气健脾汤治疗儿童慢性咳嗽（肺脾气虚型）临床研究 [硕士]: 长春中医药大学; 2014.

166. 陈志鑫, 郝瑞芳. 银翘散加减治疗儿童上气道咳嗽综合征50例. 河北中医. 2014;36(04):547-8.

167. 陈萍. 补肺汤加减治疗难治性慢性咳嗽58例. 江西中医药. 2014;45(02):43-4.

168. 黄慧, 金宁, 张继友. 止嗽散联合三拗汤加减治疗慢性咳嗽的疗效和安全性分析. 中国实用医药. 2014;9(18):182-3.

169. 黄艳春, 邓小全. 半夏泻心汤治疗慢性咳嗽42例. 中国中医药现代远程教育. 2014;12(20):145+9.

170. 邱宏. 使用参芪白术散合泻白散治疗小儿慢性咳嗽的效果分析. 当代医药论丛. 2015;13(17):25-6.

171. 仇年芳, 童蓓丽. 滋阴活血法治疗阴虚血瘀型慢性咳嗽30例. 中国中西医结合耳鼻咽喉科杂志. 2015;23(06):466-7.

172. 唐存祥, 胡一莉. 利肺汤治疗慢性咳嗽40例疗效观察. 浙江中医杂志. 2015;50(01):31.

173. 戴蓓, 蔡值, 董莹, 葛潮. 加味止嗽散治疗慢性咳嗽26例疗效观察. 湖南中医杂志. 2015;31(07):42-3.

174. 雷小婷, 侯从岭. “冬病夏治”穴位贴敷疗法治疗慢性咳嗽临床研究. 中医学报. 2015;30(12):1738-40.

175. 廖颖钊, 赵丽, 王璋. 益肺祛邪止咳方治疗儿童慢性咳嗽60例疗效观察. 河北中医. 2015;37(10):1487-8.

176. 李聪敏, 张江, 李静, 张会凯. 芪冬润肺方治疗气阴亏虚型慢性咳嗽疗效观察. 河北医药. 2015;37(02):214-5.

177. 李聪敏, 张江, 李静, 张会凯. 芪冬润肺汤治疗气阴亏虚型慢性咳嗽39例临床观察. 河北中医. 2015;37(02):174-6.

178. 房志家. 止嗽散加减合苓甘五味姜辛汤治疗慢性咳嗽的临床价值分析. 大家健康(学术版). 2015;9(23):41-2.

179. 王建华, 黄菊萍. 加味六君子汤治疗慢性咳嗽疗效观察. 基层医学论坛. 2015;19(25):3519-20.

180. 王燕. 二陈汤联合四君子汤加味治疗46例小儿慢性咳嗽疗效观察. 光明中医. 2015;30(09):1901-2.

181. 王亚红, 严海君. 穴位贴敷在小儿慢性咳嗽中的疗效分析. 中国医药指南. 2015;13(31):194.

182. 汪凤仙, 曹明祥, 赵萍. 四逆散加减治疗胃食管反流病引起的慢性咳嗽临床观察. 新中医. 2015;47(12):44-6.

183. 田爱平, 赵辉, 张丽. 膏方联合穴位贴敷治疗慢性咳嗽的疗效观察. 中医药导报. 2015;21(17):61-2.

184. 程远魁. 半夏泻心汤化裁治疗慢性咳嗽临床疗效观察. 深圳中西医结合杂志. 2015;25(24):65-6.

185. 叶焰, 金华伟, 李俐. 从痰论治对改善痰浊阻肺型慢性咳嗽生活质量的影响. 新中医. 2015;47(07):44-5.

186. 邢燕如, 陆玉廷, 刘婷, 刘秋海, 李炜. 通窍宣肺汤为主治疗儿童上气道咳嗽综合征48例. 浙江中医杂志. 2015;50(08):599.

187. 胡冬梅. 玄参升麻汤治疗小儿慢性咳嗽的临床探析. 中国卫生标准管理. 2015;6(07):243-4.

188. 吕秀梅. 自拟祛风止嗽颗粒加减治疗慢性咳嗽342例. 光明中医. 2015;30(09):1899-900.

189. 吴学敏. 止嗽散加减合苓甘五味姜辛汤治疗88例慢性咳嗽的疗效分析. 中医临床研究. 2015;7(01):81-2.

190. 孙贺. 平肝止咳汤治疗久咳(肝阳上亢，肺失清肃证)的临床观察 [硕士]: 长春中医药大学; 2015.

191. 张文汉, 赵鼎铭, 许友慧, 车爱红, 宋宏安. 麻杏石甘汤加减联合咳宁膏贴敷治疗小儿慢性咳嗽340例疗效观察. 河北中医. 2015;37(05):730-2.

192. 张小瑾. 病炎清15号方治疗外寒内饮型PNDS的临床研究 [硕士]: 广州中医药大学; 2015.

193. 张佩月. 止咳验方治疗慢性咳嗽疗效观察. 山西中医. 2015;31(04):29-30.

194. 张昊, 谢永曼. 自拟润肺化痰膏治疗慢性咳嗽60例临床观察. 中国社区医师. 2015;31(10):81+3.

195. 纪燕, 卢云, 张亚玲. 参苓白术散联合西药治疗慢性咳嗽随机平行对照研究. 实用中医内科杂志. 2015;29(03):127-8.

196. 赵华铭. 养阴清肺汤合止嗽散治疗慢性咳嗽87例. 河南中医. 2015;35(09):2272-3.

197. 闫旭明. 二陈汤加味治疗慢性咳嗽痰浊犯肺型50例临床观察. 中医临床研究. 2015;7(04):60-2.

198. He ML, Wu MD, Deng YX, Li HJ. Effect observation of magnetic bead pressurized auricular point combined with Reyanbao of traditional Chinese medicine in treatment of acute attack of chronic bronchitis. China modern medicine [zhong guo dang dai yi yao]. 2015;22(24):114‐6, 9.

199. 高鸽. 桑杏玉女煎治疗慢性咳嗽（胃阴不足，燥邪干肺）的临床研究 [硕士]: 长春中医药大学; 2016.

200. 廖卓越. 加味六君子汤配合罗红霉素胶囊对慢性咳嗽患者止咳作用的临床疗效评价. 抗感染药学. 2016;13(06):1352-4.

201. 李海霞. 皮内针调理肝肺法治疗慢性特发性咳嗽的临床研究 [硕士]: 北京中医药大学; 2016.

202. 毛玉安. 祛风散化裁治疗慢性咳嗽48例疗效观察. 湖南中医杂志. 2016;32(12):47-8.

203. 徐琴, 吴峰, 徐宪韬. 蓝芩口服液治疗青年上气道咳嗽综合征引起的慢性咳嗽临床观察. 河北中医. 2016;38(10):1511-4.

204. 王丹, 杨秀娟, 王远照, 祁波. 穴位贴敷联合顺尔宁治疗小儿慢性咳嗽肺虚邪恋型50例. 陕西中医药大学学报. 2016;39(01):78-80.

205. 王芸. 旋覆泻心止咳汤应用于胃食管反流性咳嗽的效果观察和护理. 当代护士(上旬刊). 2016(09):33-4.

206. 王靖, 杜文娟. 滋阴止咳汤治疗小儿慢性咳嗽临床研究. 河北中医. 2016;38(05):689-92.

207. 王丽娟, 周笑梅. 苓甘五味姜辛汤联合西药治疗慢性咳嗽疗效观察. 新中医. 2016;48(12):32-3.

208. 王丽军, 臧力学. 中西医结合治疗痰热蕴阻型慢性咳嗽疗效观察. 山西中医. 2016;32(02):29-30.

209. 于向艳, 马蕴蕾, 耿立梅, 马红梅, 闫红倩, editors. 小青龙汤加减治疗慢性咳嗽患者的临床效果分析2016.

210. 刁志宁. 中医中药治疗慢性咳嗽临床观察. 中西医结合心血管病电子杂志. 2016;4(36):153+6.

211. 彭天托, 李秀兰, 梁兆铭. 中西医结合治疗慢性咳嗽疗效观察. 实用中医药杂志. 2016;32(05):463-4.

212. 何帆. 泻白散加减治疗痰热郁肺型小儿慢性咳嗽的疗效观察 [硕士]: 广州中医药大学; 2016.

213. 胡芳, 赵立杰, 郭军英. 三拗汤加减联合四子散外敷治疗儿童慢性咳嗽临床观察. 四川中医. 2016;34(05):92-4.

214. 花佳佳, 张玲燕. 宣肺平肝汤治疗不明原因慢性咳嗽临床研究. 陕西中医药大学学报. 2016;39(02):32-4.

215. 严炜. 止嗽散治疗慢性咳嗽临床观察与分析. 人人健康. 2016(14):80.

216. 乔明飞. 苓甘五味姜辛汤合止嗽散加减治疗慢性咳嗽临床分析. 光明中医. 2016;31(11):1529-30.

217. 刘宏, editor 小柴胡汤治疗社区慢性咳嗽的临床观察. 中华中医药学会全科医学分会成立大会暨2016年学术年会; 2016; 中国上海.

218. 刘文彦. 自拟宁肺汤治疗小儿慢性咳嗽肺脾气虚型的临床研究 [硕士]: 北京中医药大学; 2016.

219. 刘娥. 辛防宣肺汤合孟鲁司特钠治疗儿童上气道咳嗽综合征（风痰郁肺证）临床疗效观察 [硕士]: 青岛大学; 2016.

220. 刘慧兰. 尉中民教授学术思想与临床经验总结及培土生金法治疗小儿慢性咳嗽的疗效评价研究 [博士]: 北京中医药大学; 2016.

221. 刘晓莉, 谢守勇. 理中汤加减治疗慢性咳嗽临床观察. 中医临床研究. 2016;8(17):107-8.

222. 吕莉. 痰喘宁合剂治疗痰湿蕴肺型小儿慢性咳嗽临床疗效观察 [硕士]: 首都医科大学; 2016.

223. 喻群. 鼻肺咳方治疗上气道咳嗽综合征湿热型的临床研究 [硕士]: 广州中医药大学; 2016.

224. 张玲燕, 花佳佳, 乔楠. 宣肺平肝法治疗不明原因慢性咳嗽的临床研究. 陕西中医药大学学报. 2016;39(06):60-2.

225. 张丽辉, 张永昌, 王兵. 养阴清肺法治疗慢性咳嗽38例. 光明中医. 2016;31(02):239-41.

226. 杨昆, 黄芹, 孟晓露. 新制苍耳子散治疗小儿上气道咳嗽综合征40例. 广西中医药. 2016;39(02):48-9.

227. 杨旋芳. 穴位敷贴治疗慢性咳嗽的疗效观察与护理. 中国药物经济学. 2016;11(10):137-9.

228. 荆晶. 诃子止咳散治疗小儿慢性咳嗽110例. 实用中医药杂志. 2016;32(03):217-8.

229. 蒋诗媛, 杨焕彪. 温肺化饮方配合药线点灸治疗慢性咳嗽临床观察. 广西中医药. 2016;39(04):22-4.

230. 贾建营, 赵海云. 二陈汤化裁治疗儿童上气道咳嗽综合征30例疗效观察. 中医临床研究. 2016;8(04):102-3.

231. 邹佳丽. 强效止咳糖浆对阴虚肺热型慢性咳嗽的临床疗效观察 [硕士]: 湖北民族学院; 2016.

232. 陈丽兰, 林艺娟, 陈阿兰, 郑丽萍. 中药穴位贴敷治疗儿童感染后咳嗽86例. 福建中医药. 2016;47(02):61.

233. 陈庆海. 止嗽散联合西药治疗儿童慢性咳嗽54例临床观察. 中医儿科杂志. 2016;12(06):48-51.

234. 韩俊. 加味二陈汤治疗小儿痰湿蕴肺型慢性咳嗽的疗效分析. 实用医技杂志. 2016;23(11):1239-40.

235. 唐晓媛, 曾省都, 刘惟优, 陈国峰, 袁小亮, 饶运帷. 厚朴排气合剂治疗胃食管反流性咳嗽的疗效观察. 世界华人消化杂志. 2017;25(05):448-51.

236. 雷颖, 陈庆海. 止嗽散治疗小儿慢性咳嗽的临床观察. 实用中西医结合临床. 2017;17(03):128-9+31.

237. 利广平. 加味三拗汤联合纳米穴位贴穴位敷贴治疗小儿慢性咳嗽的疗效分析. 吉林医学. 2017;38(10):1887-9.

238. 林志成. 清风汤治疗慢性咳嗽合并过敏性鼻炎（风痰阻肺证）临床观察 [硕士]: 长春中医药大学; 2017.

239. 李华, 李颉, 郭婷婷. 三拗汤合六君子汤化裁治疗小儿慢性咳嗽临床研究. 河南中医. 2017;37(07):1255-7.

240. 凡全女. 止咳降气汤治疗98例慢性咳嗽的效果研究. 实用临床医药杂志. 2017;21(17):185-6.

241. 胥媛. 养阴清肺汤联合孟鲁司特治疗55例小儿支原体肺炎后慢性咳嗽的效果评价. 河南医学研究. 2017;26(01):144-5.

242. 宋明达. 玄贝温胆汤治疗寒地儿童上气道咳嗽综合征（湿热内蕴证）的临床疗效观察 [硕士]: 黑龙江中医药大学; 2017.

243. 申燕华, 钱叶长, 马伟, 危蕾, 张善芳. 苓甘五味姜辛汤联合西医疗法治疗慢性咳嗽的临床研究. 中国医药导刊. 2017;19(12):1337-9.

244. 王列, 马铁明, 于本性, 马帅, 沈红岩. 基于“冬病夏治”论穴位贴敷结合小儿推拿治疗小儿慢性咳嗽. 辽宁中医杂志. 2017;44(12):2623-5.

245. 王首, 赵珊珊, 赵敏, 阮婉芬, 蒙来成, 苏春荀, et al. 调理中气法治疗岭南地区小儿慢性咳嗽60例临床观察. 中医儿科杂志. 2017;13(02):36-8.

246. 魏瑜. 祛风宣肺、养阴润燥法治疗慢性咳嗽临床研究. 长春中医药大学学报. 2017;33(04):587-9.

247. 章羽. 针刺配合拔罐治疗小儿慢性咳嗽临床研究. 中医学报. 2017;32(06):932-5.

248. 周雪冰, 张海生, 李为. 玄参升麻汤联合复方甲氧那明治疗儿童慢性咳嗽的临床疗效观察. 湖南师范大学学报(医学版). 2017;14(04):4-6.

249. 朱婴. 自拟益气补肺汤治疗肺脾气虚型慢性咳嗽临床观察. 四川中医. 2017;35(06):166-8.

250. 朱腾西. 清燥救肺汤加减联合西药治疗慢性咳嗽49例. 光明中医. 2017;32(02):268-70.

251. 沈丽萍, 吕祺美. 止嗽散加减辨位治疗小儿慢性咳嗽45例. 中国中医药科技. 2017;24(04):531-2.

252. 何建. 健脾止咳汤治疗慢性咳嗽临床研究. 中国社区医师. 2017;33(22):105+7.

253. 刘玉. 分析小青龙汤加减治疗慢性咳嗽的临床效果. 内蒙古中医药. 2017;36(02):6.

254. 刘会智, 刘丽, 陈也. 苓甘五味姜辛汤联合复方甲氧那明治疗慢性咳嗽临床研究. 西南国防医药. 2017;27(06):585-8.

255. 刘签兴, 李晓洁. 李士懋教授治疗慢性咳嗽验案1例. 天津中医药. 2017;34(09):581-2.

256. 张立山, 周绍忠, 张顺利, 田爱萍, 陈一川, 秦东风, et al. 柴朴汤治疗191例慢性咳嗽临床研究. 中国中医药信息杂志. 2017;24(04):29-31.

257. 张俊, 倪伟, 吴淑红. 咳喘六味合剂治疗慢性咳嗽33例. 光明中医. 2017;32(05):650-1.

258. 张海霞. 自拟清肺化痰汤治疗小儿肺炎支原体感染后慢性咳嗽（痰热壅肺证）临床疗效观察 [硕士]: 安徽中医药大学; 2017.

259. 罗智聪, 胡小毅, 范春红. 研究疏风降气止咳汤对改善慢性咳嗽患者气道高反应性的效果. 罕少疾病杂志. 2017;24(02):30-2.

260. 许正利. 补肺汤联合西药治疗老年慢性咳嗽临床研究. 新中医. 2017;49(03):41-3.

261. 谢波. 中药颗粒治疗慢性咳嗽的临床效果分析. 内蒙古中医药. 2017;36(16):41.

262. 赖丽. 冬贝止咳胶囊治疗慢性咳嗽疗效观察. 内蒙古中医药. 2017;36(15):17.

263. 郑全秀. 清阳肃肺汤治疗慢性咳嗽（肠胃湿热、肺失清肃证）的临床观察 [硕士]: 长春中医药大学; 2017.

264. 韦跃文, 李志宏. 养阴清肺汤合止嗽散治疗慢性咳嗽疗效观察. 现代养生. 2017(20):155-6.

265. 韩雪. 雷火灸治疗小儿慢性咳嗽51例的效果分析. 中国继续医学教育. 2017;9(10):180-1.

266. 韩旭东. 止嗽散加减治疗慢性咳嗽的有效性分析. 中医临床研究. 2017;9(34):92-3.

267. 马文建, 鲁玉芬, 刘永新, 李克. 小儿肺咳颗粒治疗儿童感染后咳嗽的疗效与作用机制. 中国实验方剂学杂志. 2017;23(14):204-9.

268. 齐瑞. 桑杏汤加味治疗小儿咳嗽的临床观察 [硕士]: 第四军医大学; 2017.

269. 郭龙. 半夏泻心汤加减治疗慢性咳嗽的临床疗效研究. 中国卫生标准管理. 2018;9(24):80-2.

270. 涂媚. 苓甘五味姜辛汤合止嗽散加减治疗慢性咳嗽临床观察. 实用中医药杂志. 2018;34(01):15-6.

271. 梁玉玲. 刺四缝配合健脾化痰法治疗小儿慢性咳嗽临床观察. 新中医. 2018;50(01):119-22.

272. 黎永明. 中药穴位贴敷治疗痰湿蕴肺型慢性咳嗽疗效观察. 四川中医. 2018;36(03):188-9.

273. 李瑞婷, 齐瑞, 罗剑锋, 王爱丽, 张月萍. 桑杏二陈汤与孟鲁司特钠治疗儿童感染后咳嗽的临床对照研究. 现代生物医学进展. 2018;18(09):1705-8.

274. 李瑞丽. 止嗽散加减辨位治疗小儿慢性咳嗽42例分析. 实用中西医结合临床. 2018;18(10):42-4.

275. 李燕坪. 乌梅丸加减治疗风盛挛急型慢性咳嗽的临床观察 [硕士]: 广州中医药大学; 2018.

276. 李叶雨. 黄连温胆汤治疗小儿胃食管反流性咳嗽（痰热内蕴，兼食积证）的临床疗效观察 [硕士]: 成都中医药大学; 2018.

277. 李华成. 采用小柴胡汤加减治疗难治性慢性咳嗽的临床效果评析. 当代医药论丛. 2018;16(07):223-4.

278. 徐华智, 李莉. 苓甘五味姜辛汤合止嗽散加减治疗慢性咳嗽的临床疗效. 深圳中西医结合杂志. 2018;28(13):64-5.

279. 王评, 彭晓洪, 黄亚秀, 黄永莲. 麦朴汤治疗慢性咳嗽临床研究. 中医药临床杂志. 2018;30(09):1678-81.

280. 尹璐, 秦芳, 李祖长. 养阴清肺汤合止嗽散对慢性咳嗽的效果观察. 湖北中医药大学学报. 2018;20(02):64-7.

281. 田亚楠. 西药联合益气补肺汤治疗慢性咳嗽42例疗效观察. 中国民族民间医药. 2018;27(19):99-100.

282. 曹秋梅. 咳宁合剂治疗咳嗽变异型哮喘临床研究 [硕士]: 成都中医药大学; 2018.

283. 周凤华. 止嗽散治疗慢性咳嗽的临床观察. 中国继续医学教育. 2018;10(21):143-4.

284. 佟洋洋. 清上宣肺汤治疗咳嗽（湿热干肺，肺失宣肃证）的临床观察 [硕士]: 长春中医药大学; 2018.

285. 彭明浩, 宋桂华. 麦门冬汤加减治疗小儿慢性咳嗽经验. 中国中西医结合儿科学. 2018;10(04):358-60.

286. 何明礼, 谢一民, 卢长国, 马嘉忆, 李莎. 用二陈汤合三子养亲汤加减对47例痰湿蕴肺型慢性咳嗽患者进行治疗的效果探讨. 当代医药论丛. 2018;16(18):129-31.

287. 何燕娜. 针刺四缝穴联合穴位贴敷治疗小儿慢性咳嗽效果观察. 临床医学. 2018;38(07):75-6.

288. 何增. 清燥救肺汤加减方治疗小儿感染后咳嗽风燥伤肺证的临床观察 [硕士]: 黑龙江中医药大学; 2018.

289. 侯瀚翔, 刘归. 升阳益胃汤加减联合止嗽散加味治疗慢性咳嗽的临床效果. 河南医学研究. 2018;27(22):4114-5.

290. 丛方方, 朱姬莲. 沙参麦冬汤合芍药甘草汤治疗肺炎支原体感染后小儿慢性咳嗽的分析. 健康之路. 2018;17(01):199-200.

291. 严飞飞, 魏建玲, 何晓娜. 玉屏风散治疗小儿慢性咳嗽临床应用及效果评估. 亚太传统医药. 2018;14(07):188-9.

292. 冯木兰, 庄玲玲, 陈水凤. 生物陶瓷热敷袋穴位热敷治疗风寒袭肺证成人慢性咳嗽患者的效果观察. 齐齐哈尔医学院学报. 2018;39(24):2911-3.

293. 刘欢. 辛夷清肺饮加减治疗小儿上气道咳嗽综合征（痰热内蕴证）的临床观察 [硕士]: 山西中医药大学; 2018.

294. 华浩昌. 穴位贴敷疗法联合小儿推拿疗法治疗小儿慢性咳嗽的效果及安全性. 中外医学研究. 2018;16(28):173-4.

295. 卢桢, 余进. 用养阴清肺汤合止嗽散治疗慢性咳嗽的效果探究. 当代医药论丛. 2018;16(05):12-4.

296. 孙雪莲. 泻肺降浊汤治疗咳嗽（大肠瘀积，肺经伏火证）的临床研究 [硕士]: 长春中医药大学; 2018.

297. 孙亚飞. 益气补肺汤联合西药治疗慢性咳嗽43例临床观察. 中国民族民间医药. 2018;27(17):103-5.

298. 张健, 赵小明. 三拗汤合六君子汤化裁治疗小儿慢性咳嗽的临床疗效. 临床医学研究与实践. 2018;3(26):117-8.

299. 张磊. 半夏泻心汤治疗咳嗽（脾虚胃热证）的临床研究 [硕士]: 长春中医药大学; 2018.

300. 张诗竹. 黄龙止咳颗粒治疗慢性咳嗽（阴虚肺燥证）随机对照临床研究 [硕士]: 成都中医药大学; 2018.

301. 杨晓颖. 经络诊察法配合穴位埋针治疗慢性咳嗽临床观察. 中外医学研究. 2018;16(18):3-5.

302. 罗琴. 中药竹罐配合穴位贴敷治疗慢性咳嗽的疗效观察. 当代护士(上旬刊). 2018;25(12):144-5.

303. 苏叶芳. 止嗽散加减治疗慢性咳嗽的临床疗效观察. 世界最新医学信息文摘. 2018;18(91):151.

304. 许早荣, 郑爱红. 九仙散合止嗽散加减治疗慢性咳嗽体会. 新疆中医药. 2018;36(01):83-4.

305. 贺建军. 探讨分析止嗽散合三拗汤加减对慢性咳嗽的临床疗效和用药安全. 中医临床研究. 2018;10(07):111-3.

306. 贺红安. 麻杏二三汤治疗小儿慢性咳嗽痰湿阻肺型的临床观察 [硕士]: 山西中医药大学; 2018.

307. 赖瑜. 加味苓甘五味姜辛汤联合三伏贴穴位贴敷治疗慢性咳嗽的临床观察. 中国民间疗法. 2018;26(09):34-5.

308. 陆晋. 止嗽散合三拗汤加减治疗慢性咳嗽的效果及安全性分析. 当代医药论丛. 2018;16(17):191-3.

309. 陈金凤. 针刺自主神经相应区域治疗慢性咳嗽的临床研究 [硕士]: 长春中医药大学; 2018.

310. 陈水凤, 冯木兰, 杨惠生. 生物陶瓷穴位热敷对慢性咳嗽患者生活质量的影响. 中国卫生标准管理. 2018;9(21):186-8.

311. 陈凯欣. 穴位埋线与针刺治疗肺脾两虚型慢性咳嗽对照研究 [硕士]: 广州中医药大学; 2018.

312. 鲁军. 止嗽降气汤治疗慢性咳嗽的临床分析. 中外医疗. 2018;37(13):179-80+83.

313. 黄明朝. 分析止嗽降气汤治疗慢性咳嗽的临床应用效果. 中国卫生标准管理. 2018;9(23):92-4.

314. 黄波贞. 半夏厚朴汤合麦门冬汤治疗慢性咳嗽60例临床观察. 湖南中医杂志. 2018;34(11):51-3.

315. 黄莹. 柴朴汤治疗小儿上气道咳嗽综合征（痰气互结证）临床观察 [硕士]: 广州中医药大学; 2018.

316. 龚建齐, 邹广华. 通窍止咳汤联合标准桃金娘油肠溶胶囊治疗鼻后滴漏综合征致慢性咳嗽43例. 环球中医药. 2018;11(11):1823-5.

317. 高作良, 高梅. 止嗽散合三拗汤治疗慢性咳嗽患儿的疗效及对炎症因子、免疫功能的影响. 中医临床研究. 2019;11(36):31-3.

318. 霍健, 刘焕, 苏兴利, 赵妍, 李春燕. 参苓白术散加减对肺癌术后慢性咳嗽的疗效及相关机制研究. 世界中医药. 2019;14(09):2300-4.

319. 郭景瑞. 沙参麦冬汤治疗小儿慢性咳嗽肺阴亏虚证的临床效果. 临床医学研究与实践. 2019;4(06):97-8+122.

320. 霍巧维. 探讨冬病夏治穴位贴敷疗法治疗慢性咳嗽肺脾阳虚证临床疗效. 中国社区医师. 2019;35(29):91-2.

321. 董雪妍, 林军, 陈艳, 陈慧敏, 唐虹, 周湖来. 基于《咳嗽的诊断与治疗指南(2015)》治疗慢性咳嗽临床观察. 湖北中医杂志. 2019;41(07):29-31.

322. 杜光瑜, 章潇迪, 张建玉, 邱薇, 秦小刚, 王武强. 中西医结合治疗儿童过敏性咳嗽疗效观察. 实用中医药杂志. 2019;35(05):582-3.

323. 梁安琦. 海派推拿治疗风邪伏肺型小儿感染后咳嗽疗效观察 [硕士]: 广州中医药大学; 2019.

324. 廖福建, 汪莎莎. 小柴胡汤联合香砂六君子汤治疗小儿慢性咳嗽疗效观察. 临床合理用药杂志. 2019;12(10):103-4.

325. 李延梅. 穴位敷贴治疗慢性咳嗽的疗效观察与护理探讨. 中国农村卫生. 2019;11(24):62-3.

326. 林丽荣, 莫火秀. 穴位贴治疗肺结核慢性咳嗽的效果与护理. 临床医药文献电子杂志. 2019;6(81):125-6.

327. 李炜. 养阴清肺汤联合孟鲁司特治疗小儿支原体肺炎致慢性咳嗽临床研究. 新中医. 2019;51(01):70-3.

328. 孟晓雨. 自拟益气止咳汤治疗小儿慢性咳嗽（肺脾气虚型）的临床观察 [硕士]: 长春中医药大学; 2019.

329. 潘玉梅, 杨若莹, 瞿慧. 加味二陈汤治疗小儿痰湿蕴肺型慢性咳嗽的临床疗效观察. 临床医药文献电子杂志. 2019;6(90):26.

330. 方红苏. 平肝宁肺方治疗慢性咳嗽（肝火犯肺证）的临床研究 [硕士]: 成都中医药大学; 2019.

331. 范亚丽. 自拟通窍止咳汤治疗儿童上气道咳嗽综合征临床疗效评价. 辽宁中医药大学学报. 2019;21(05):126-9.

332. 付茹, 李永刚, 解亚军, 苟宝龙. 自拟清肺汤联合西药治疗慢性咳嗽疗效观察. 西部中医药. 2019;32(05):94-6.

333. 徐春霞. 百合固金汤加减治疗虚火灼肺型慢性咳嗽的临床疗效. 内蒙古中医药. 2019;38(10):15-6.

334. 王宏伟, 李国利. 自拟清热宣肺祛风利咽方治疗慢性咳嗽风咳证疗效观察. 四川中医. 2019;37(09):77-80.

335. 王磊. 宣肺化湿止咳汤治疗湿热郁肺型CVA的临床观察 [硕士]: 江西中医药大学; 2019.

336. 王立娟. 益气养阴法治疗慢性咳嗽52例疗效观察. 世界最新医学信息文摘. 2019;19(49):177+89.

337. 王文佳. 蒿芩清胆汤加味治疗慢性咳嗽（胆胃失和证）的临床观察 [硕士]: 长春中医药大学; 2019.

338. 王倩. 小青龙汤合九仙散加减方治疗慢性咳嗽疗效分析. 临床医药文献电子杂志. 2019;6(87):10.

339. 王春辉. 孟鲁司特钠联合皮内针穴位埋置治疗儿童慢性咳嗽效果观察. 中国医学前沿杂志(电子版). 2019;11(10):79-81.

340. 于尚多. 穴位贴敷联合小儿推拿治疗小儿非特异性慢性咳嗽临床研究 [硕士]: 长春中医药大学; 2019.

341. 魏琦. 寒咳宁加味联合鼻腔冲洗治疗上气道咳嗽综合征（风寒恋肺型）的临床观察 [硕士]: 江西中医药大学; 2019.

342. 任航, 刘芳. 化痰通窍汤治疗小儿上气道咳嗽综合征临床观察. 山西中医. 2019;35(08):48-9+60.

343. 岑杨成. 三拗汤合六君子汤化裁治疗小儿慢性咳嗽的临床研究. 名医. 2019(07):266.

344. 周坤先, 梁燕婷, 李芳, 章锦红. 穴位贴敷结合推拿治疗慢性咳嗽疗效观察. 实用中医药杂志. 2019;35(10):1259-60.

345. 朱淑然. 四君子汤合姜辛味夏治疗儿童感染后咳嗽的临床研究 [硕士]: 广州中医药大学; 2019.

346. 周泳. 曾氏润肺止咳汤治疗肺阴亏虚型慢性咳嗽疗效初探 [硕士]: 广州中医药大学; 2019.

347. 周婷. 通窍止咳汤治疗儿童上气道咳嗽综合征（痰湿蕴肺型）临床疗效观察 [硕士]: 贵州中医药大学; 2019.

348. 朱晓萌. 滋阴止咳汤联合孟鲁司特治疗支原体肺炎后慢性咳嗽患儿50例. 中西医结合研究. 2019;11(06):300-1.

349. 曾丽君, 赖婵. 止咳降气汤治疗慢性咳嗽的临床疗效研究. 深圳中西医结合杂志. 2019;29(10):57-9.

350. 刘玉. 天麻二陈汤加减治疗儿童感染后风痰恋肺型咳嗽临床观察 [硕士]: 云南中医药大学; 2019.

351. 刘中友, 陈丽华. 苓甘五味姜辛汤联合甲氧那明胶囊治疗慢性咳嗽疗效观察. 实用中医药杂志. 2019;35(02):204-5.

352. 刘华建. 麻龙止咳汤对CVA患者（阴虚肺燥型）PEF变异率、FeNO水平影响及临床疗效研究 [硕士]: 成都中医药大学; 2019.

353. 刘晓玲, 李娜. 半夏泻心汤加减治疗慢性咳嗽的效果评价. 名医. 2019(11):237.

354. 吕彩虹, 彭暾. 加味香砂六君子颗粒治疗小儿肺脾气虚型慢性咳嗽的临床研究. 内蒙古中医药. 2019;38(03):16-7.

355. 张玲. 加味桔梗汤治疗慢性咳嗽50例. 实用中医药杂志. 2019;35(12):1455-6.

356. 张成旭. 中医综合诊疗方案对慢性咳嗽儿童生活质量的影响 [硕士]: 上海中医药大学; 2019.

357. 张翠云, 陈小燕. 自拟益气补肺汤和复方甲氧那敏胶囊治疗肺脾气虚型慢性咳嗽的效果对比. 内蒙古中医药. 2019;38(04):12-3.

358. 张艳云. 半夏泻心汤加减联合西药治疗慢性咳嗽临床疗效观察. 四川中医. 2019;37(01):109-12.

359. 杨胜利. 活血化瘀法治疗慢性咳嗽临床观察 [硕士]: 湖北中医药大学; 2019.

360. 罗本华, 梁薇, 郭雅雯, 潘云云, 李文康, 郭柳婷. “围大暑立秋节气隔姜火龙灸”治疗肺脾气虚型慢性咳嗽21例疗效观察. 大众科技. 2019;21(02):59-60+4.

361. 谢玉贤, 黄晓莉. 自拟祛风止嗽颗粒治疗慢性咳嗽临床效果评价. 深圳中西医结合杂志. 2019;29(05):49-50.

362. 陈丹, 陈春梅, 黄沂, 吕文欣, 宁余音. 食疗联合雷火灸治疗阳虚质慢性咳嗽疗效观察. 广西中医药大学学报. 2019;22(01):14-7.

363. 陈明, 周继朴, 王玉光. 加减三仁汤配合孟鲁斯特钠治疗鼻后滴流综合征所致湿热型慢性咳嗽疗效及对小气道功能及气道高反应性的影响. 现代中西医结合杂志. 2019;28(33):3672-6+92.

364. 黄葵好. 麻杏石甘汤加减治疗呼吸道感染后慢性咳嗽的临床效果观察. 中外医学研究. 2019;17(19):118-9.

365. 康芳. 三拗汤合六君子汤治疗小儿慢性咳嗽的效果分析. 中国冶金工业医学杂志. 2020;37(03):266-7.

366. 郭新雪, 叶琛琛, 王勤, 王敏华, 吴佳妮, 江明月. 冬病夏治穴位贴敷治疗小儿慢性咳嗽疗效观察. 实用妇科内分泌电子杂志. 2020;7(08):135-6+8.

367. 匡奕亮, 叶雄誉, 刘健芳, 卢利员. 苓甘五味姜辛汤合止嗽散加减治疗慢性咳嗽临床观察. 中外医学研究. 2020;18(26):28-30.

368. 李亚梅, 王永军, 王文第. 裴氏止咳化痰膏穴位贴敷辅助治疗小儿慢性咳嗽110例临床观察. 中医儿科杂志. 2020;16(06):89-92.

369. 李华, 李颉, 李飞飞. 三拗汤合六君子汤治疗儿童慢性咳嗽（痰湿阻肺证）的疗效观察. 上海医药. 2020;41(21):4-6+57.

370. 李颉, 戎芬, 李华, 何丽, 丁惠玲, 李战, et al. 化痰祛风方治疗小儿呼吸道感染后慢性咳嗽(痰湿证)的临床研究. 上海中医药杂志. 2020;54(10):62-7.

371. 孟令一, 王磊, 王少廉, 赵静. 旋覆夏麻芎芍草汤加减治疗慢性咳嗽临床观察. 上海中医药大学学报. 2020;34(03):18-21.

372. 范娟娟, 李光, 章冬娥. 三拗疏风汤治疗嗜酸粒细胞性支气管炎的疗效观察. 中国医药科学. 2020;10(04):43-5.

373. 扶佳玲. 健脾止咳汤治疗慢性咳嗽的效果评价. 当代医药论丛. 2020;18(03):4-5.

374. 傅大治, 盛丽先. 名中医经验方治疗儿童喉源性咳嗽30例临床观察. 海峡药学. 2020;32(04):126-7.

375. 沙艳萍. 半夏厚朴汤合麦门冬汤治疗慢性咳嗽的效果探析. 当代医药论丛. 2020;18(12):186-7.

376. 石明杰, 王梁敏, 唐玲, 张艳霞, 马建岭, 刘静, et al. 穴位贴敷联合耳穴压丸治疗老年慢性咳嗽痰湿蕴肺证疗效观察. 中西医结合护理(中英文). 2020;6(07):61-4.

377. 徐传藩, 吴怀敏, 徐盼. 苓甘五味姜辛汤合止嗽散加减治疗慢性咳嗽的疗效观察. 内蒙古中医药. 2020;39(04):57-8.

378. 徐玥瑾, 张学智. 旋杏二陈汤治疗风痰阻肺型慢性咳嗽临床研究. 中华中医药杂志. 2020;35(11):5877-9.

379. 易科成, 郑远方. 沙参玉竹汤加减治疗慢性咳嗽的临床效果. 中国当代医药. 2020;27(12):16-9.

380. 伍晓瑛, 李佩芳. 通调三焦法治疗慢性咳嗽疗效观察. 山西中医. 2020;36(09):50-1.

381. 王宏杰, 杨之藻. 中药煮散剂治疗儿童慢性咳嗽（痰热壅肺证）30例临床观察. 国医论坛. 2020;35(05):26-8.

382. 王敏, 尹惠婷, 张旭. 自组方与穴位贴敷结合治疗肺炎支原体感染后慢性咳嗽临床研究. 四川中医. 2020;38(07):100-3.

383. 王仕奎. 补肺止咳汤治疗儿童慢性咳嗽. 中医学报. 2020;35(07):1555-9.

384. 王皖洁. 苏子降气汤加减治疗慢性咳嗽的回顾性研究. 中医临床研究. 2020;12(15):27-8.

385. 田红妮, 邵嫱. 三拗汤联合四子散外敷治疗儿童慢性咳嗽的效果观察. 实用临床医药杂志. 2020;24(07):50-2.

386. 朱莎, 肖波. 陈夏六君汤加减治疗慢性咳嗽的临床观察. 内蒙古中医药. 2020;39(12):19-20.

387. 朱吕群. “风户”穴导气针法对慢性咳嗽的临床疗效观察 [硕士]: 南京中医药大学; 2020.

388. 何玉玲. 射麻止嗽汤治疗小儿慢性咳嗽（风伏肺络型）的临床疗效观察 [硕士]: 山西中医药大学; 2020.

389. 杭文璐, 赵杰, 马雷, 李彦, 王倩. 苏黄止咳胶囊治疗慢性咳嗽临床观察. 光明中医. 2020;35(23):3678-81.

390. 兰森宁. 止嗽散加减治疗肺结核慢性咳嗽的效果观察. 中国冶金工业医学杂志. 2020;37(02):205-6.

391. 刘文可. 小儿推拿治疗儿童上气道咳嗽综合征的临床疗效观察 [硕士]: 北京中医药大学; 2020.

392. 吴杰妍, 凌钦亮. 小五味子汤、布地奈德联合推拿治疗感染后慢性咳嗽患儿的临床疗效. 世界中西医结合杂志. 2020;15(10):1942-5+60.

393. 孙红彦, 魏春雨. 润燥止嗽汤佐治慢性咳嗽的疗效及对血清炎性因子的影响. 国医论坛. 2020;35(02):40-2.

394. 张永丽, 张俊臻. 中西药合用治疗小儿鼻后滴漏综合征致慢性咳嗽临床观察. 实用中医药杂志. 2020;36(11):1416-7.

395. 张颖颖, 陈昆. 柴黄颗粒联合妥洛特罗治疗对小儿慢性咳嗽患者的临床疗效. 中成药. 2020;42(12):3372-4.

396. 杨孟, 冉志玲. 自拟桂芍理肺汤治疗风痰恋肺型儿童上气道咳嗽综合征48例. 江西中医药. 2020;51(06):38-40.

397. 杨秀丽. 黄龙止咳胶囊治疗慢性咳嗽（风盛挛急证）的随机双盲临床研究 [硕士]: 成都中医药大学; 2020.

398. 杨赛芬, 刘海英. 滋阴养肺止咳汤联合阿奇霉素治疗儿童慢性咳嗽的疗效分析. 中国中医药科技. 2020;27(01):154-5.

399. 许婷. 明清吴门医派咳病用药分析及加味叶氏养胃汤治疗阴虚型慢性支气管炎的临床研究 [硕士]: 南京中医药大学; 2020.

400. 谭弘珍, 郑远方. 小柴胡汤加减治疗慢性咳嗽的临床效果. 中国当代医药. 2020;27(32):152-4.

401. 贾晓妍. 养阴清肺汤加减治疗肺炎支原体感染后咳嗽（阴虚证）的临床疗效观察 [硕士]: 辽宁中医药大学; 2020.

402. 赖春林. 苓甘五味姜辛汤合止嗽散加减治疗慢性咳嗽的效果观察. 临床合理用药杂志. 2020;13(14):52-3.

403. 赵影, 袁林, 练志明. 杏苏散合小柴胡汤加减治疗慢性咳嗽的临床观察. 中国实用医药. 2020;15(12):151-3.

404. 邹凤春. 滋阴止咳汤治疗小儿慢性咳嗽临床研究. 临床医药文献电子杂志. 2020;7(05):95.

405. 姜永红, 姜之炎, 肖臻, 李俊霞, 顾静雯, 毛黎明, et al. PC-QoL问卷评价中药联合耳穴治疗对1～6岁慢性咳嗽患儿生活质量的影响. 现代中西医结合杂志. 2021;30(05):457-62.

406. 季锡林. 止嗽散加减治疗慢性咳嗽的临床效果. 临床合理用药杂志. 2021;14(23):67-9.

407. 金倩. 安胃宁肺止嗽汤治疗胃食管反流性咳嗽痰热上扰型的临床观察 [硕士]: 云南中医药大学; 2021.

408. 唐琳, 周淑娟, 兰珊. 王氏保赤丸治疗儿童反流性咳嗽的临床观察. 上海医药. 2021;42(15):16-8.

409. 唐明杰. 苓甘五味姜辛汤联合止嗽散加减治疗慢性咳嗽40例临床观察. 中医临床研究. 2021;13(18):29-32.

410. 李姣锋, 吕会茹, 申绯翡, 黄晓利. 润肺平喘汤加减联合孟鲁司特钠治疗小儿肺脾气虚型慢性咳嗽临床研究. 中华中医药学刊. 2021;39(11):243-6.

411. 李冬梅. 咽咳舒喷雾剂治疗咽喉炎所致慢性咳嗽的临床疗效观察 [硕士]: 云南中医药大学; 2021.

412. Lyu YR, Kim KI, Yang C, Jung SY, Kwon OJ, Jung HJ, et al. Efficacy and Safety of Ojeok-San Plus Saengmaek-San for Gastroesophageal Reflux-Induced Chronic Cough: a Pilot, Randomized, Double-Blind, Placebo-Controlled Trial. Frontiers in pharmacology. 2022;13.

413. 李迎春. 苓甘五味姜辛汤合止咳散加减治疗慢性咳嗽的疗效及安全性分析. 中国农村卫生. 2021;13(04):5-6.

414. 李泽嘉. 培土生金法在小儿慢性咳嗽推拿治疗中的应用效果观察 [硕士]: 广西中医药大学; 2021.

415. 武鹏. 补肺汤联合西药治疗慢性咳嗽的效果及对咳嗽积分的影响. 黑龙江中医药. 2021;50(05):165-6.

416. 方俊成, 黎业鹏, 王志伟. 三参二梗汤联合西药治疗气阴两虚型慢性咳嗽的临床效果观察. 中国中医药科技. 2021;28(01):145-7.

417. 范国梅. 止嗽散加减治疗慢性咳嗽的效果及安全性分析. 北方药学. 2021;18(08):158-9.

418. 付啸峰, 高海利, 谢壁元. 清燥救肺汤加减治疗气阴两虚型非小细胞肺癌慢性咳嗽的效果. 中国医学创新. 2021;18(20):97-101.

419. 司徒婉玲, 甄慧慈, 余剑佩, 陈小群. 小儿推拿法联合穴位按摩在小儿支原体肺炎感染后慢性咳嗽护理中的应用. 中外医学研究. 2021;19(18):146-8.

420. 史彦超, 孙晓旭. 三拗汤加味佐用四子散联合氨溴特罗口服液在慢性咳嗽患儿中的应用效果. 中国民康医学. 2021;33(08):76-8.

421. 徐响瑜, 蔡淑琴, 俞素青. 自拟宣肺汤合六君子汤治疗慢性咳嗽的临床观察. 中国中医药科技. 2021;28(03):469-70.

422. 邵成良. 润肺通络汤联合孟鲁司特钠治疗小儿肺炎支原体感染后慢性咳嗽46例. 中国中医药科技. 2021;28(01):134-5.

423. 倪晨阳. 清热化痰、宣肺通窍法治疗小儿上气道咳嗽综合征的临床研究 [硕士]: 南京中医药大学; 2021.

424. 王宏静. 清肝汤治疗慢性咳嗽（肝火犯肺证）的临床疗效观察 [硕士]: 湖南中医药大学; 2021.

425. 王步青, 刘家宏, 郝瑞春, 张树君. 镇肝熄风汤加减联合复方甲氧那明胶囊治疗风盛挛急型慢性咳嗽的临床观察. 中国民间疗法. 2021;29(02):78-81.

426. 汪淑琴, 陈一柳, 张成旭, 姜永红. 小儿干咳方联合耳穴贴压治疗儿童慢性咳嗽风邪伤肺证疗效观察. 中华中医药杂志. 2021;36(12):7505-8.

427. 王依依, 迟振海. 老十针针刺结合肌筋膜触发点松解治疗慢性咳嗽案. 中国民间疗法. 2021;29(21):118-9.

428. 王洪, 马媛媛, 王莉, 孟牛安, 陈广坤. 中药煮散剂健脾益气治疗小儿肺脾气虚型慢性咳嗽的临床观察. 中国中西医结合儿科学. 2021;13(06):526-9.

429. 任永魁, 王秀兰. 清肺汤联合冬病夏治穴位贴敷治疗慢性咳嗽临床观察. 中国药业. 2021;30(20):93-5.

430. 庄秋凤. 小儿消积止咳颗粒联合阿奇霉素治疗小儿肺炎支原体感染后慢性咳嗽疗效观察. 实用中医药杂志. 2021;37(05):819-20.

431. 丁雪阳. 止咳清肃汤治疗小儿感染后咳嗽（风热犯肺证）的临床疗效观察 [硕士]: 山东中医药大学; 2021.

432. 周敏. 胃食管反流性咳嗽的中药复方治疗观察 [硕士]: 江西中医药大学; 2021.

433. 朱晓倩, 龚春颖. 中医穴位贴敷护理技术在慢性咳嗽中的应用效果. 医学信息. 2021;34(17):190-2.

434. 周静. 小儿肺咳颗粒与盐酸氨溴索糖浆治疗小儿慢性咳嗽临床对比研究. 新中医. 2021;53(05):120-3.

435. 崔延英, 赵岩, 刘小虎. 自拟祛风止嗽汤治疗慢性咳嗽的效果评价. 中国现代药物应用. 2021;15(16):185-7.

436. 冯奕超, 王志强. 止咳散加减联合盐酸氨溴索颗粒在慢性咳嗽治疗中的应用分析. 现代诊断与治疗. 2021;32(06):850-1.

437. 刘卿. 杏苏散加减治疗小儿感染后咳嗽风寒恋肺证的临床观察 [硕士]: 天津中医药大学; 2021.

438. 刘艳. “温中降逆法”联合质子泵抑制剂治疗胃食管反流性咳嗽（中虚气逆证）的临床观察 [硕士]: 成都中医药大学; 2021.

439. 卫旭鹏. 小儿推拿配合刮痧治疗上气道咳嗽综合征（湿热内蕴型）的临床研究 [硕士]: 长春中医药大学; 2021.

440. 孙亚娟. 止嗽散加减治疗慢性咳嗽的疗效及对血清指标的影响. 中国民间疗法. 2021;29(21):61-3.

441. 张佳奇. 参冬养阴清金汤治疗小儿慢性咳嗽（阴虚肺热型）90例临床疗效观察 [硕士]: 长春中医药大学; 2021.

442. 张媚, 吴婷, 曾丽燕, 张奕星. 益气养阴祛风汤、小儿推拿联合盐酸西替利嗪治疗气阴两虚型小儿慢性咳嗽临床研究. 新中医. 2021;53(24):111-4.

443. 张灏, 许鹤龄, 吕红, 陆兵, 舒君, 钱星佳, et al. 甘麦大枣汤联合栀子豉汤治疗更年期女性慢性咳嗽. 吉林中医药. 2021;41(10):1319-21.

444. 张苏贤, 潘宝峰, 张伟伟, 杨克敏, 许敏, 韩虹宇, et al. 定咳汤干预难治性慢性咳嗽有效性的单臂纵向研究. 上海医药. 2021;42(08):19-22.

445. 张静怡. 呼出气一氧化氮水平与慢性咳嗽中医证候的相关性研究及祛风咳敏方的疗效观察 [硕士]: 天津中医药大学; 2021.

446. 栾岚, 印有亮, 韩雪娇. 西药联合通窍止咳汤治疗鼻后滴漏综合征致慢性咳嗽的临床研究. 当代医学. 2021;27(07):8-11.

447. 谌婷婷, 芦盛贞. 刮痧联合热敏灸治疗风寒袭肺型慢性咳嗽的临床观察. 中国当代医药. 2021;28(14):162-5.

448. 谢珊, 唐颖丽, 余清萍, 何霞. 中药包穴位热敷辅助治疗成人慢性咳嗽风寒袭肺证疗效观察. 四川中医. 2021;39(01):73-6.

449. 郎秋雯, 李佩芳. 背俞穴走罐治疗慢性咳嗽的临床观察. 中国民间疗法. 2021;29(08):58-60.

450. 陆璐. 穴位贴敷联合综合护理对门诊慢性咳嗽患者的干预效果. 中西医结合护理(中英文). 2021;7(08):45-8.

451. 陈丹, 陈琳. 二陈汤合小青龙汤加减对儿童慢性咳嗽的临床疗效观察. 中医临床研究. 2021;13(04):99-101.

452. 陈一柳, 汪淑琴, 张成旭, 吕佳佳, 赵晓阳, 包春秀, et al. 小儿湿咳方联合穴位敷贴治疗儿童慢性湿性咳嗽临床研究. 广州中医药大学学报. 2021;38(03):468-74.

453. 陈子翔. 以“风户”穴为主通督导气针刺法治疗风邪伏肺型慢性咳嗽的临床研究 [硕士]: 南京中医药大学; 2021.

454. 陈军红, 张阳阳, 韩思. 玉屏风颗粒辅助治疗小儿慢性咳嗽临床研究. 新中医. 2021;53(09):13-6.

455. 黄文婷. 通元针法治疗肺肾两虚型慢性咳嗽临床疗效观察 [硕士]: 广州中医药大学; 2021.

456. 黄森. 止嗽散加味联合常规西药治疗慢性咳嗽患者的临床效果. 中国民康医学. 2021;33(19):85-7.

457. 黄雪梅. 温肺降气止咳汤合穴位贴敷治疗寒邪滞肺型慢性咳嗽临床研究 [硕士]: 江西中医药大学; 2021.

458. 黄娟. 桑杏苏芩汤合六君子汤加减治疗儿童感染后咳嗽肺脾气虚证的临床观察 [硕士]: 甘肃中医药大学; 2021.

459. 郭芬芳, 武青. 麻杏二三汤加减联合推拿治疗儿童慢性咳嗽痰湿阻肺证的疗效及对患儿肺功能、炎症因子水平的影响. 海南医学. 2022;33(08):1024-6.

460. 戴兴龙, 吴玄珠. 自拟止咳养肺汤治疗儿童慢性咳嗽40例. 中国中医药科技. 2022;29(03):506-8.

461. 王芳, 侯秋雨, 李明心. 参苓白术散加味治疗肺癌根治术后慢性咳嗽临床疗效及对患者炎性因子及免疫功能的影响. 四川中医. 2022;40(05):76-9.

462. 崔佳宾, 李金鹏, 张东仓, 苟早平. 苓甘五味姜辛汤联合三子养亲汤加减治疗慢性咳嗽的临床疗效. 甘肃科技. 2022;38(04):99-101.

463. 何翠红, 黄沂, 宁余音, 梁群, 陈静. 壮医通路灸改善儿童慢性咳嗽的效果研究. 护理研究. 2022;36(07):1267-9.

464. 冯思, 黄璐. 小儿止咳方联合穴位敷贴辅助治疗儿童慢性咳嗽痰湿蕴肺证38例临床观察. 中医儿科杂志. 2022;18(04):62-6.

465. 冯超, 王秀兰. 清燥救肺汤加减治疗慢性咳嗽临床研究. 中国药业. 2022;31(13):103-6.

466. 刘恒源, 段元元, 李光熙. 透皮外用温散酊治疗慢性咳嗽临床观察. 世界中西医结合杂志. 2022;17(03):576-9.

467. 张暮盈, 王旭红. 麻杏石甘汤加减联合复方甲氧那明胶囊治疗慢性咳嗽临床观察. 山西中医. 2022;38(04):13-5.

468. 张子燕, 秦胜娟. 泻肺化痰汤联合孟鲁司特钠治疗非特异性慢性咳嗽痰热壅肺型临床研究. 实用中医药杂志. 2022;38(06):974-5.

469. 罗建峰, 陈梅兰. 调肺运脾小儿推拿法联合二陈汤加减治疗痰湿蕴肺型小儿慢性咳嗽临床研究. 新中医. 2022;54(14):176-9.

470. 谢波. 疏风止嗽汤治疗慢性咳嗽的临床疗效. 中国现代医生. 2022;60(02):131-3+7.

471. 赵一粒, 拓晓萍. 宣肺运脾汤治疗儿童慢性咳嗽(痰湿蕴肺型)的疗效及对患儿炎症因子、免疫功能的影响. 海南医学. 2022;33(12):1559-62.

472. 陈磊. 苏黄止咳胶囊结合推拿治疗小儿慢性咳嗽疗效观察. 实用中医药杂志. 2022;38(07):1096-7.

473. 黄雪梅, 万丽玲. 温肺降气止咳汤联合穴位贴敷治疗慢性咳嗽40例临床观察. 中国民族民间医药. 2022;31(03):114-8.

474. 龚洁秋. 参苓白术散合玉屏风散加减辅助治疗小儿慢性咳嗽肺脾气虚证40例临床观察. 中医儿科杂志. 2022;18(04):53-7.
