# Supplementary material for: Research status of east Asian traditional medicine treatment for chronic cough: A scoping review
Source: PLoS One. 2024 Feb 8;19(2):e0296898. doi: 10.1371/journal.pone.0296898 (PMC10852285; doi:10.1371/journal.pone.0296898)
Supplement: S4 Appendix — (DOCX) [file pone.0296898.s004.docx]

**S4 Appendix. Cause of cough of the included studies according to the age**

| Cause of cough | Children | | Adults | | Both | | Not reported | | Total | |
| --- | --- | --- | --- | --- | --- | --- | --- | --- | --- | --- |
|  | N | % | N | % | N | % | N | % | N | % |
| UACS | 29 | 16.9% | 11 | 4.8% | 5 | 7.5% | 0 | 0.0% | 45 | 9.5% |
| CVA | 2 | 1.2% | 6 | 2.6% | 2 | 3.0% | 0 | 0.0% | 10 | 2.1% |
| GERD | 3 | 1.7% | 13 | 5.6% | 3 | 4.5% | 0 | 0.0% | 19 | 4.0% |
| Chronic bronchitis | 0 | 0.0% | 4 | 1.7% | 1 | 1.5% | 0 | 0.0% | 5 | 1.1% |
| Eosinophilic bronchitis | 0 | 0.0% | 2 | 0.9% | 0 | 0.0% | 0 | 0.0% | 2 | 0.4% |
| Post-infection | 37 | 21.5% | 4 | 1.7% | 4 | 6.0% | 0 | 0.0% | 45 | 9.5% |
| Nonspecific | 7 | 4.1% | 15 | 6.5% | 6 | 9.0% | 0 | 0.0% | 28 | 5.9% |
| Unexplained | 1 | 0.6% | 5 | 2.2% | 1 | 1.5% | 0 | 0.0% | 7 | 1.5% |
| Mixed | 13 | 7.6% | 17 | 7.4% | 5 | 7.5% | 1 | 25.0% | 36 | 7.6% |
| Others* | 1 | 0.6% | 10 | 4.3% | 0 | 0.0% | 0 | 0.0% | 11 | 2.3% |
| Not reported | 79 | 45.9% | 144 | 62.3% | 40 | 59.7% | 3 | 75.0% | 266 | 56.1% |
| **Total** | **172** | **100.0%** | **231** | **100.0%** | **67** | **100.0%** | **4** | **100.0%** | **474** | **100.0%** |

*including bronchiectasis, interstitial pulmonary fibrosis, allergic cough, angiotensin-converting enzyme inhibitors-induced cough, psychogenic cough, airway hyperresponsiveness, chronic obstructive pulmonary disease, cough after lung cancer surgery, pulmonary tuberculosis, chronic sore throat, etc.

Abbreviations. CVA, cough variant asthma; GERD, gastroesophageal reflux disease; UACS, upper airway cough syndrome.
